# Identification of an mRNA isoform switch for HNRNPA1 in breast cancers

**DOI:** 10.1038/s41598-021-04007-y

**Published:** 2021-12-27

**Authors:** Murat Erdem, İbrahim Ozgul, Didem Naz Dioken, Irmak Gurcuoglu, Sezen Guntekin Ergun, Rengul Cetin-Atalay, Tolga Can, Ayse Elif Erson-Bensan

**Affiliations:** 1 Department of Biological Sciences, Middle East Technical University (METU), Dumlupinar Blv No: 1 Universiteler Mah., Cankaya, Ankara, 06800 Turkey; 2 Cancer Systems Biology Laboratory, CanSyL, Graduate School of Informatics, Middle East Technical University, 06800 Ankara, Turkey; 3 Department of Computer Engineering, Middle East Technical University (METU), Dumlupinar Blv No: 1 Universiteler Mah, Ankara, 06800 Turkey; 4 Present Address: Department of Medical Biology, Hacettepe University, Ankara, Turkey

**Keywords:** Breast cancer, Molecular biology, Transcription

## Abstract

Roles of HNRNPA1 are beginning to emerge in cancers; however, mechanisms causing deregulation of HNRNPA1 function remain elusive. Here, we describe an isoform switch between the 3′-UTR isoforms of *HNRNPA1* in breast cancers. We show that the dominantly expressed isoform in mammary tissue has a short half-life. In breast cancers, this isoform is downregulated in favor of a stable isoform. The stable isoform is expressed more in breast cancers, and more HNRNPA1 protein is synthesized from this isoform. High HNRNPA1 protein levels correlate with poor survival in patients. In support of this, silencing of HNRNPA1 causes a reversal in neoplastic phenotypes, including proliferation, clonogenic potential, migration, and invasion. In addition, silencing of HNRNPA1 results in the downregulation of microRNAs that map to intragenic regions. Among these miRNAs, miR-21 is known for its transcriptional upregulation in breast and numerous other cancers. Altogether, the cancer-specific isoform switch we describe here for HNRNPA1 emphasizes the need to study gene expression at the isoform level in cancers to identify novel cases of oncogene activation.

## Introduction

Advances in RNA-sequencing (RNA-seq) methods revealed more than 90% of human genes to produce tissue-specific alternative mRNA isoforms, which add substantial complexity to the transcriptome in higher vertebrates^1–3^. Potential mechanisms that cause and coordinate this isoform diversity are; alternative use of transcription start sites, alternative splicing, and alternative polyadenylation^4,5^. The resulting mRNA isoforms may or may not share the same 5′UTRs (untranslated regions), coding sequences (CDSs), or 3′UTRs^6,7^. As a result, differences in the CDSs and UTRs may alter mRNA stability, cellular localization, and translation rate, leading to changes in protein functions or levels^3^. Hence, the type of isoforms and their relative expression levels become important variables for gene expression regulation in normal and disease states. As we begin to appreciate the depth and extent of isoform diversity in the transcriptome, new findings point out changes in relative ratios of mRNA isoforms, referred to as isoform switches. Accumulating evidence highlights the role of isoform switches with biological impact in normal tissues and diseases, including cancer^8–10^. For example, a shorter isoform of *BCL*, *BCL-XS*, activates apoptosis and is a tumor suppressor, whereas the longer isoform, *BCL-XL,* is an oncogene blocking apoptosis^11^. The ratio of these two oppositely functioning mRNA isoforms is altered in cancers in favor of the longer isoform, enhancing survival^12^. Similarly, expression of a *BRAF* (B-Raf proto-oncogene) isoform that lacks the RAS binding domain promotes resistance to *BRAF* inhibition in a group of melanoma patients^4,13^.

High throughput efforts provide further evidence that pathologic shift of isoform ratios may disrupt protein–protein interactions in different cancers^14^. Hence, the discovery of cancer-specific isoform switches holds promising potential as diagnostic biomarkers and therapy targets^9,15^.

This study describes a cancer-specific isoform switch for a versatile RNA binding protein (RBP), HNRNPA1. We took a combinatorial in silico and in vitro approach to identify and verify the isoform switch between 3′-end isoforms of *HNRNPA1*. We show that *HNRNPA1* isoforms with different 3′UTRs have different half-lives and the cancer-specific isoform switch contributes to increased HNRNPA1 protein abundance in breast cancer cells. We also provide new insight into HNRNPA1 function in indirectly modulating the expression of intragenic microRNAs such as miR-21, a well-known oncomiR. These results emphasize the importance of gene expression analysis at the isoform level in cancer cells, revealing unknown oncogene activation cases with biological impact.

## Results

### Isoform level analysis

**Figure 1:**
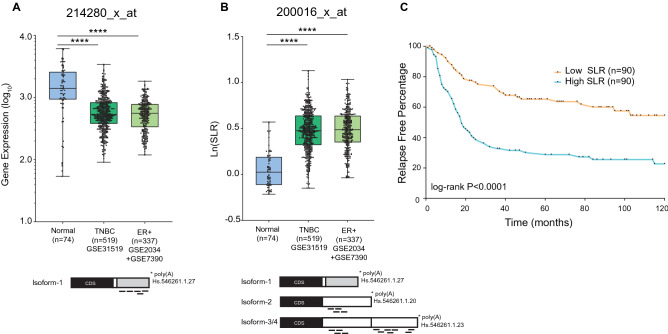
Isoform level expression of *HNRNPA1*. (**A**) *HNRNPA1* Isoform-1 expression in breast cancer patients compared with normal breast tissue (****p < 0.0001, unpaired t-test) in GSE31519, GSE2034, and GSE7390 datasets. Positions of Affymetrix distal probes (214280_x_at) recognizing only Isoform-1 are shown. (**B**) SLR values (Isoform 2 + 3 + 4/Isoform 3 + 4) of *HNRNPA1* in breast cancer patients compared with normal breast tissue (****p < 0.0001, unpaired t-test) in GSE31519, GSE2034, and GSE7390 datasets. Positions of Affymetrix distal probes (200016_x_at) recognizing Isoform-2,3,4 are shown. (**C**) High expression (of Isoform-2, 3, and 4) correlates with poor relapse-free survival in TNBC patients. GSE31519 dataset was grouped according to the top 25% (High) and bottom 25% (Low) expressing patients. Expression values are based on proximal probes of 200016_x_at, excluding levels of Isoform-1. Hazard ratio (95% CI) is 2.709 (log-rank p < 0.0001).

In a targeted screen for *HNRNPA1* expression in breast cancers, we re-analyzed GSE31519, GSE2034, GSE7390 datasets using APADetect, an algorithm to detect isoform level expression differences based on differential poly(A) site usage^16,17^. We analyzed data sets for probe-level differences based on the positions of poly(A) sites. Probes proximal to poly(A) sites generally recognize all isoforms, whereas distal probes recognize longer isoforms. Ratios of proximal to distal probe sets were calculated for normal and cancer samples. Significant changes in the signal intensities were reported as SLR ((Short + Long)/Long ratio). In breast cancer patients (n = 856), independent from tumor type, we observed significant downregulation (p < 0.0001) of an *HNRNPA1* isoform that ends with a distal poly (A) site on the gene locus (Hs.546261.1.27) (Fig. 1A). The downregulated isoform (hereon called Isoform-1) has a different 3’UTR than other isoforms of *HNRNPA1* due to the inclusion of two non-coding terminal exons (exon 12, 13). Only the distal probes of 214280_x_at (Affymetrix probe set ID) recognize Isoform-1 specifically.

On the contrary, the SLR of other isoforms was high in patients compared to normal breast tissue (Fig. 1B). The Isoform-2, 3, 4 were co-detected (but could not be distinguished) and quantified by the distal probes of the 200016_x_at probe set as these isoforms have the same terminal exon (exon 11). These isoforms either have a shorter 3’UTR (Isoform-2) or a longer 3’UTR (Isoform-3, 4). Overall, breast cancer patients have an increased isoform ratio (Short + Long isoforms)/Long isoforms) for *HNRNPA1.* The ratio shift was significant; however, we could not determine individual expression levels because both proximal and distal probes recognize multiple isoforms. Interestingly, increased expression of isoforms detected by proximal probes of 200016_x_at correlates with patient relapse times in the GSE31519 cohort (Fig. 1C). These data suggested that isoforms are differentially expressed in patients.

To begin confirming the in silico patient data, we first validated the 3′-ends of isoforms by 3′-RACE, cloning, and sequencing (Supplementary Figs. S1–S3). Next, we tested breast cancer cell lines (n = 18) and a panel of breast cancer patient cDNAs (n = 25) by RT-qPCR (Supplementary Fig. S4). We detected an increased ratio of isoforms compared to Isoform-1 in 40% of breast cancer cell lines and approximately 90% of patient samples. However, the culprit of microarray data and RT-qPCR was the use of probes and primers recognizing more than one isoform. To delineate isoform-specific expression, we turned to RNA-seq data of Genotype-Tissue Expression (GTEx) and TCGA datasets. We compared the expression of isoforms in GTEx normal tissue samples to TCGA tumor samples using UCSC Xena, which allows comparison of the two datasets^18–20^.

**Figure 2:**
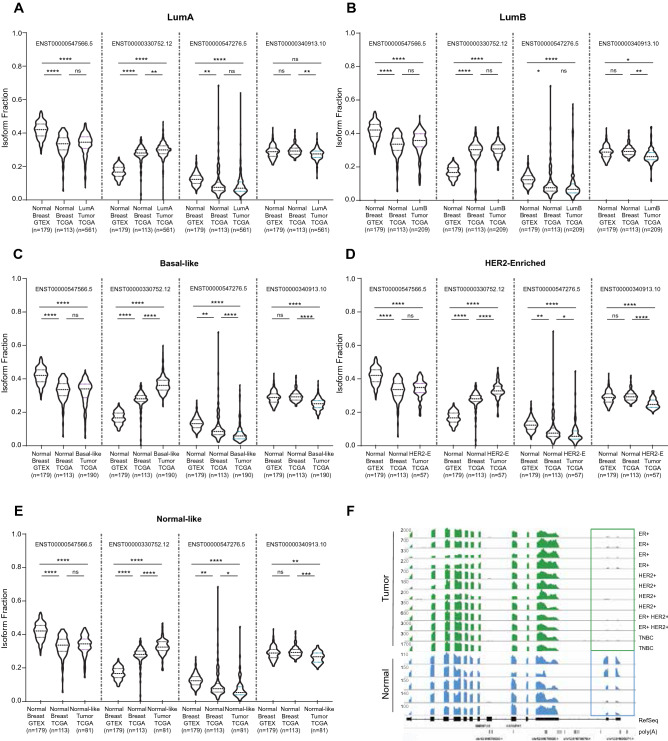
Isoform fractions of *HNRNPA1* isoforms (Isoform-1, 2, 3, 4) in normal breast (GTEX), normal adjacent tissue (TCGA), and in Luminal A (**A**), Luminal B (**B**), Basal-like (**C**), HER2-enriched (**D**), Normal-like breast cancers (**E**) in the TCGA dataset (*p < 0.05, **p < 0.01, ***p < 0.001, ****p < 0.0001, one-way ANOVA, Tukey's HSD), (**F**) Representative single-cell RNA-seq data for normal cell types and in breast cancer cells (GSE75688, GSE113197). The boxed area shows the reads from the exons unique to Isoform-1.

Surprisingly, Isoform-1, which is low in breast cancer patients (Fig. 1A), is the dominant isoform in normal mammary tissue (ENST00000547566.1) (Fig. 2). Isoform-2 (ENS ENST00000330752.12) has a short 3′UTR, and it is overexpressed in Luminal A, Luminal B, HER2 enriched, basal-like, and normal-like breast cancers compared to GTEx normal mammary tissue. Expression of Isoform-3 (ENST00000547276.5) is further decreased in all breast cancer subtypes, despite not being very abundant in normal breast tissue. Isoform-4 (ENST00000340913.10) is also downregulated in all subtypes compared to adjacent normal or GTEx normal tissue. Isoform-4 also has a long 3′UTR identical to Isoform-3 but has an exon-8 insertion (156 bp) which does not alter the reading frame.

Notably, the expression levels of some isoforms in the matched peritumor tissue of breast cancer patients were in an intermediate state compared with the normal tissues in the GTEx dataset. This pattern suggests that the adjacent normal tissue may be distinct from healthy tissue and the tumor, as was also reported in other studies^21^. The isoform switch was also evident in single-cell RNA-sequencing data of normal mammary cells compared with breast cancer cells (GSE113197, GSE75688) (Fig. 2F).

With these results, the reason behind SLR change in breast cancers (Fig. 1B) became apparent. Higher SLR was due to the upregulation of Isoform-2 and downregulation of other isoforms.

### Isoform switch and HNRPA1 protein levels

**Figure 3:**
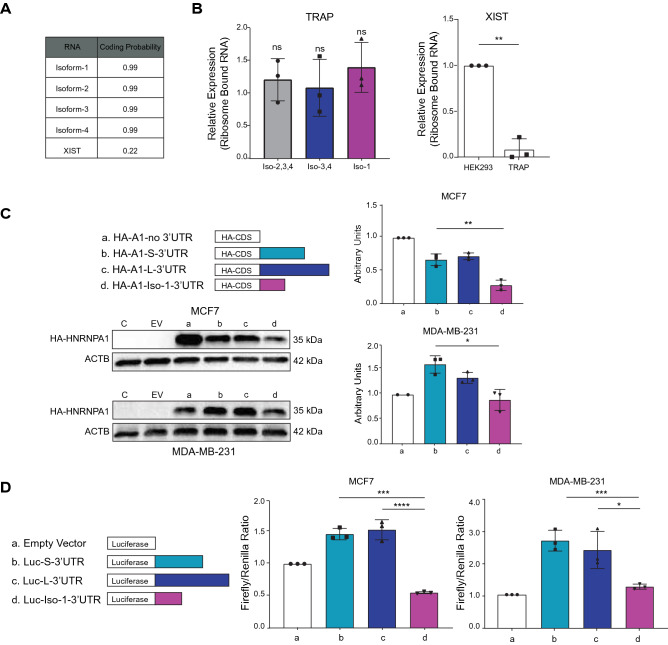
Translation of *HNRNPA1* isoforms. (**A**) Coding potentials of *HNRNPA1* isoforms determined by CPC2 are shown. *XIST* is a non-coding RNA. (**B**) TRAP in EGFP-L10A expressing HEK293 cells. Levels of TRAP RNAs were normalized to their expression values in untransfected HEK293 cells to eliminate the bias of expression levels (ns: not significant, n = 3 independent RNA-IPs, one-way ANOVA, Tukey's HSD). *XIST,* a non-coding RNA, was used as a negative control (**p < 0.01; n = 3, Student’s t-test). (**C**) Cells were transiently transfected with indicated vectors, and lysates were collected. HA antibody was used to detect HNRNPA1 levels. Same blots were hybridized with ACTB antibody. The image is representative of 3 independent experiments. Graphs show densitometric quantification of bands (*p < 0.05, **p < 0.01, one-way ANOVA, Tukey's HSD), uncropped images are presented in Supplementary Fig. S13. (**D**) Different 3′UTRs were cloned downstream of the luciferase gene in the pMIR vector. Cells were transiently transfected, and *Firefly/Renilla* luciferase read-outs from the constructs were normalized to that of empty pMIR (*p < 0.05, **p < 0.01, ****p < 0.0001; n = 3 independent transfections, one-way ANOVA, Tukey's HSD).

At this point, we wanted to investigate the functional consequences of the isoform switch. However, we were surprised to find out that Isoform-1 appears as a non-coding transcript (NR_135167). The coding sequence of HNRNPA1 ends in exon 10, and all isoforms share the same stop site despite having different terminal exons (Supplementary Fig. S5). Hence to find out whether Isoform-1 is coding for a peptide, we first calculated the coding potential using the Coding Potential Calculator 2 (CPC2) algorithm^22^ and saw that it was similar to other isoforms (Fig. 3A). To verify the coding potential experimentally, we performed ribosomal affinity purification (TRAP) of translated mRNAs, performed RT-qPCR, and normalized the polysome-bound transcript levels to no-TRAP control cells. These results showed that all isoforms were associated with the immunoprecipitated polysomes. *XIST* non-coding RNA was used as a negative control (Fig. 3B).

**Figure 4:**
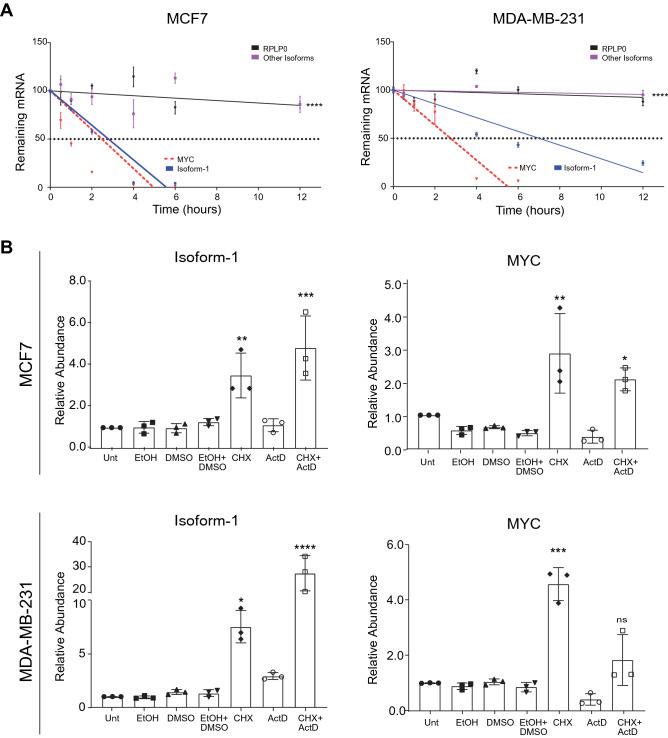
Stabilities of isoforms. (**A**) Remaining *HNRNPA1* transcripts in actinomycin D treated cells were detected by RT-qPCR. *RPLP0* as a stable mRNA and *MYC* as an unstable mRNA were used as controls (n = 3 independent treatments, ****p < 0.0001; n = 3, Student’s t-test). (**B**) Cells were treated with actinomycin D and/or cycloheximide (CHX, 100 µg/mL) for 3 h to prevent transcription and translation. EtOH (Ethanol) and DMSO are carrier controls. Cells were collected and RNA was isolated for RT-qPCR (*p < 0.05, **p < 0.01, ***p < 0.001, ****p < 0.0001, ns: not significant, n = 3 independent experiments, student’s t-test).

Next, we cloned the coding sequence of *HNRNPA1* along with the different 3′UTRs of the isoforms. Isoform-1 has a unique 3’UTR (Iso-1–3′UTR). Other isoforms share the same terminal exons, but Isoform-2 has a shorter 3′UTR (S-3′UTR) compared to the long 3′UTRs (L-3′UTR) of Isoform-3 and Isoform-4. Therefore, we tested whether these three types of 3′UTRs had different effects on protein levels. We transfected MCF7 and MDA-MB-231 cells with the HA-tagged HNRNPA1 protein expression constructs. Protein expression was detected by western blotting. Of interest, the level of HNRNPA1 protein encoded by the construct with Iso-1–3′UTR was markedly lower than the other isoforms (Fig. 3C). Because transfection efficiency could be a reason for this observation, we cloned the three different 3′UTRs downstream of a reporter gene and transiently transfected cells for a dual luciferase assay where transfection efficiencies were normalized. Here too, the luciferase reporters for the S-3′UTR and L-3′UTR had significantly higher activities than Iso-1–3′UTR in both cells (Fig. 3D). Results from the 3′UTR-reporter system, along with forced expression of HA-tagged proteins, suggested that expression of Isoform-1 correlated with lower protein levels. Since we did not see a difference in ribosome association of isoforms (Fig. 3A), we tested whether mRNA half-lives could affect HNRNPA1 protein abundance. We tested mRNA levels of *HNRNPA1* isoforms following actinomycin D treatment for 12 h to prevent new transcription. RT-qPCR results showed that Isoform-1 had a short half-life, comparable to *MYC* mRNA, well-known for its short half-life^23^ (Fig. 4A). In contrast, other isoforms were still stable after 12 h when we finalized the experiment in MCF7 and MDA-MB-231 cells. Similar decay rates were determined in MCF10A cells (non-tumorigenic mammary epithelial) (Supplementary Fig. S6).

We also treated cells with actinomycin D and cycloheximide, an inhibitor of ribosomal elongation^24^. Interestingly, cycloheximide treatment for only 3 h had a dramatic recovery effect only for Isoform 1 (> 3.5 fold in MCF7, > 7.5 fold in MDA-MB-231) (Fig. 4B, Supplementary Fig. S7). This quick recovery suggests that Isoform-1 is co-translationally degraded, as cycloheximide is also known to inhibit mRNA decay^25^.

These results indicated that the isoform switch results in differential expression of isoforms with different mRNA stabilities, affecting protein levels. However, we also tested whether 3′UTRs may regulate the localization of HNRNPA1 protein, as was suggested for a few interesting cases^26,27^. In this case, the nuclear localization of HNRNPA1 was independent of 3′UTR sequences of isoforms (Supplementary Fig. S8).

**Figure 5:**
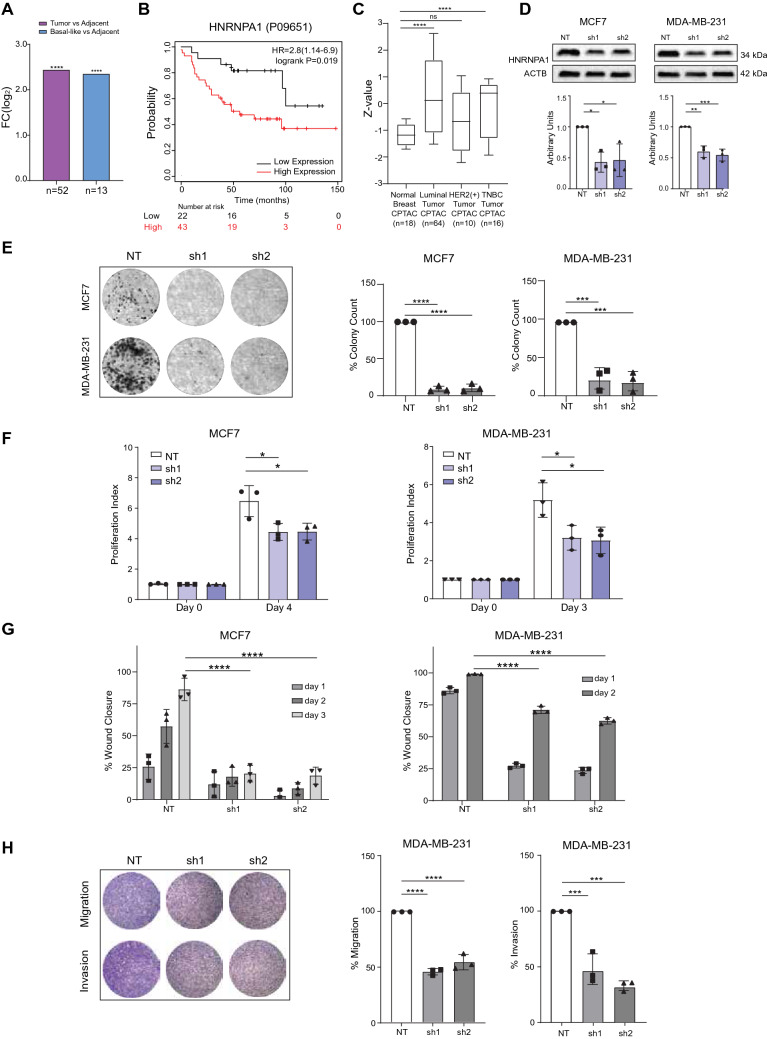
HNRNPA1 protein levels and function in breast cancers. (**A**) Upregulation of HNRNPA1 protein in breast tumors compared with adjacent tissue pairs (n = 52) and in basal-like breast cancers (n = 13) compared with adjacent tissue. Fold change values were taken from Tang et al., study^28^ (****p < 0.0001, Wald test), (**B**) Kaplan–Meier plots were generated from the KM Plotter database (http://kmplot.com/analysis/). Plots represent the percentage of overall survival in breast cancer patients in the Tang et al., study. (HNRNPA1 protein high: red and low: black) (p = 0.019, HR = 2.8, and FDR = 50%). (**C**) HNRNPA1 protein expression in luminal, HER2 + and TNBC groups is from the CPTAC data from UALCAN (http://ualcan.path.uab.edu/analysis.html) (****p < 0.0001). Log2 Spectral count ratio values from CPTAC were first normalized within each sample profile, and then normalized across samples. Z-values represent standard deviations from the median. (**D**) Two independent clones (sh1, sh2) are shown with decreased HNRNPA1 protein levels compared to non-targeting (NT) shRNA transfected controls. The same blots were hybridized with ACTB antibody to test sample loading. The image is representative of 3 independent experiments. Graphs show densitometric quantification of bands, uncropped images are presented in Supplementary Fig. S13 (*p < 0.05, **p < 0.01, ***p < 0.001; n = 3, one-way ANOVA, Tukey's HSD). (**E**) Colony formation of NTsh and HNRNPA1sh cells after 7 or 14 days in MCF7 and MDA-MB-231 cells, respectively. Colonies were counted and analyzed using CountPHICS software (***p < 0.001, ****p < 0.0001; n = 3 independent experiments, one-way ANOVA, Tukey's HSD). (**F**) Effect of HNRNPA1 silencing on proliferation rates detected by MTT (*p < 0.05; n = 3 independent experiments, one-way ANOVA, Tukey's HSD). (**G**) Effect of HNRNPA1 silencing on wound healing property of cells. Cell layers in each well were scratched by a pipette tip. Closure of wound in each well was examined at days 0, 1, 2, and 3. (***p < 0.001 and ****p < 0.0001; n = 3, one-way ANOVA, Tukey's HSD). H. Effect of HNRNPA1 knockdown on cell migration and invasion in MDA-MB-231 cells. Cells were allowed to pass through the transwell for 15 h or Matrigel-coated membranes for 18 h (***p < 0.001 and ****p < 0.0001; n = 3 independent experiments, one-way ANOVA, Tukey's HSD).

Overall, these results showed that Isoform-1 is a rapidly degraded mRNA, possibly better regulating the protein level of HNRNPA1. This unstable isoform is low in breast cancers, whereas Isoform-2 is upregulated*.* Because this switch would indicate upregulation of HNRNPA1 protein, we were curious to investigate HNRNPA1 protein levels in patient samples. Hence, we took advantage of a quantitative liquid chromatography/mass spectrometry-based proteome analysis dataset, which used protein extracts from breast tumors and adjacent non-cancerous tissues^28^. In this dataset, HNRNPA1 protein was significantly high in 52 tumors compared to normal tissues and 13 basal-like tumors compared with normal tissue (Fig. 5A). High HNRNPA1 protein levels in these patients correlated with decreased survival, strengthening the significance of the oncogenic role of HNRNPA1 (Fig. 5B). Moreover, in an independent dataset of Clinical Proteomic Tumor Analysis Consortium (CPTAC), HNRNPA1 protein was also overexpressed in luminal, HER2+, and TNBC tumors (Fig. 5C). Of note, post-translational modifications and protein–protein interactions are likely to introduce additional layers of regulation to HNRNPA1 activity in cells.

### HNRNPA1 silenced models and intragenic miRNAs

Next, to begin addressing the biological relevance of HNRNPA1 overexpression in breast cancers, we generated stable shRNA constructs to target *HNRNPA1* expression in MCF7 and MDA-MB-231 cells (Fig. 5D) and tested these models for changes in their neoplastic phenotypes. HNRNPA1 is a versatile RNA-binding protein involved in many aspects of RNA biology, so we found a significant reversal of neoplastic phenotypes in both silencing models. We observed loss of clonogenicity (Fig. 5E), decreased proliferation (Fig. 5F), decreased motility (Fig. 5G), decreased migration and invasion capability (Fig. 5H) upon sustained silencing of HNRNPA1.

**Figure 6:**
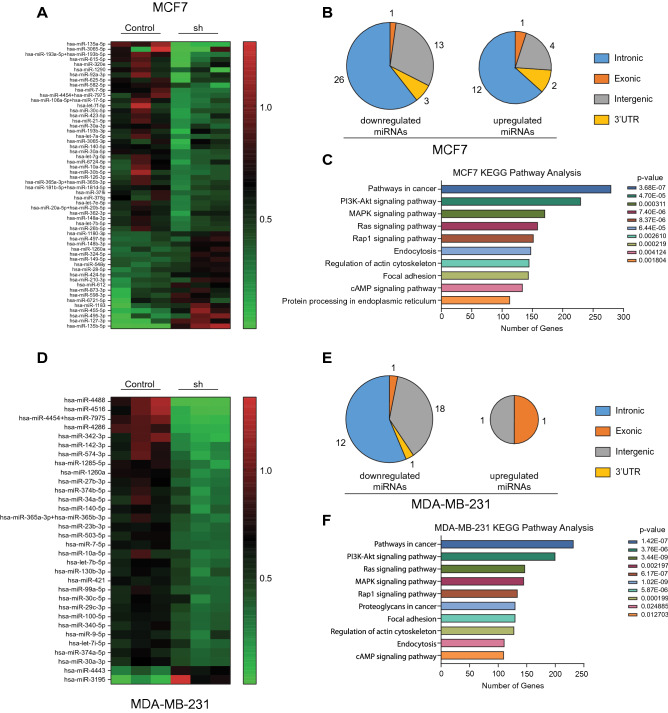
Effect of *HNRNPA1* silencing on miRNAs. (**A**) RNA isolated from HNRNPA1 silenced, and control cells were detected by NanoString miRNA panel**.** Heatmap shows miRNA expression fold changes (< 0.6 and > 1.5) in HNRNPA1sh (A1-sh) MCF7 cells. (**B**) Pie charts visualize the proportion of differentially expressed miRNAs grouped according to their genomic features in HNRNPA1sh (A1-sh) MCF7 cells (blue color for the intronic miRNAs, orange for exonic miRNAs, gray for intergenic, and yellow for miRNAs located in 3’UTRs of host genes). (**C**) Biological pathways affected by miRNAs whose expression levels were changed upon long-term *HNRNPA1* silencing in MCF7 were determined by DIANA-mirPath. (**D**) Heatmap shows miRNA expression fold changes (< 0.6 and > 1.5) in A1-sh MDA-MB-231 cells. (**E**) Pie charts visualize the proportion of differentially expressed miRNAs grouped according to their genomic features in HNRNPA1sh (A1-sh) MDA-MB-231 cells. (**F**) Biological pathways affected by HNRNPA1 regulated miRNAs in MDA-MB-231 cells.

Next, to shed light on the possible effects of HNRNPA1 activity in breast cancers, we turned to microRNAs (miRNAs) as a less explored aspect of HNRNPA1 function. HNRNPA1 has been implicated in promoting or hindering the processing steps of pri-miR-18a and pri-let-7a-1 by direct binding to loop regions^29,30^. Because HNRNPA1 has fundamental roles in RNA biology, we wanted to test whether other miRNAs would be affected by HNRNPA1 silencing. We used *HNRNPA1* silenced MCF7 and MDA-MB-231 cells (Fig. 6A,D) to screen approximately 800 miRNAs using the NanoString technology (nCounter Human miRNA assay). We detected the downregulation of mature miRNAs in HNRNPA1 silenced cells. We identified common or cell line-specific miRNAs downregulated upon HNRNPA1 silencing (Supplementary Fig. S9). A total of 43 miRNAs (70% of significantly changed miRNAs) in MCF7-sh and 32 miRNAs (94% of significantly changed miRNAs) in MDA-MB-231-sh cells were downregulated compared to NT controls. Interestingly, these miRNAs were enriched for their predicted and confirmed mRNA targets in cancer-related pathways and signaling cascades (Fig. 6C,F) which may partly explain the phenotypic changes we detected in HNRNPA1 silenced cancer cells.

**Figure 7:**
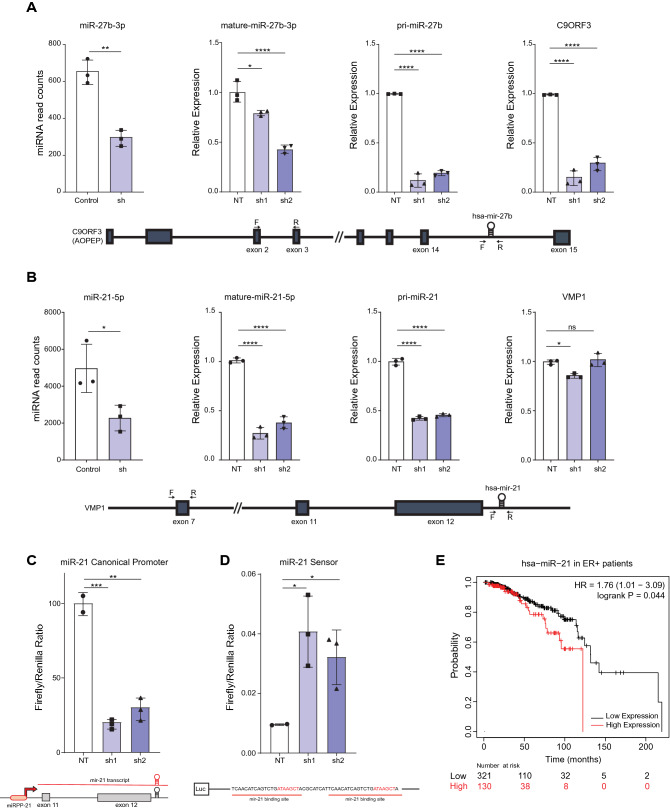
MiRNA levels in HNRNPA1 silenced cells. (**A**) miR-27b-3p count reads were decreased in HNRNPA1 silenced MDA-MB-231 cells on the NanoString miRNA panel. Mature miRNA levels in HNRNPA1 silenced MDA-MB-231 cells were detected by TaqMan miRNA PCR (n = 2 biological replicates), pri-miR-27b and host gene *C9ORF3* expression levels were quantified by RT-qPCR (*p < 0.05, **p < 0.01, ****p < 0.0001; n = 3 biological replicates, one-way ANOVA, Tukey's HSD). Gene structure of host gene, miR-27b, and primer positions are shown below the graphs. (**B**) miR-21 count reads were decreased in HNRNPA1 silenced MCF7 cells. Mature miRNA levels in HNRNPA1 silenced MCF7 cells were detected by TaqMan miRNA PCR, pri-miR-21, and host gene (*VMP1*) expression levels were quantified by RT-qPCR. (*p < 0.05, **p < 0.01, ****p < 0.0001; n = 3, one-way ANOVA, Tukey's HSD). Gene structure of host gene, miR-21 and primer positions are shown below the graphs. (**C**) Control and HNRNPA1 silenced MCF7 cells were transfected with miR-21 promoter-reporter and *Renilla* luciferase vector. Normalized luciferase activity is shown in HNRNPA1 silenced cells compared to controls (miRPP-21 is the canonical mir-21 promoter) (**p < 0.01 and ***p < 0.001 n = 3, one-way ANOVA, Tukey's HSD). (**D**) miR21 sensor activity is low in control MCF7 cells compared to HNRNPA1 silenced cells (*p < 0.05, n = 3, one-way ANOVA, Tukey's HSD). (**E**) High miR-21 expression correlates with poor overall survival in ER + breast cancer patients (TCGA) (from KM-plotter database) (logrank p = 0.044, HR = 1.76, FDR > 50%).

Notably, most downregulated miRNAs in both models were intragenic and mapped to introns of host genes (Fig. 6B,E). We reasoned that decreased expression of host genes might explain the downregulation of these miRNAs. Indeed, low miRNA read counts correlated with downregulated mature miR-27b-3p and pri-miR-27b-3p levels along with its host gene C9ORF3 on 9q22.32 in MDA-MB-231 cells upon HNRNPA1 silencing (Fig. 7A). These results suggested that the downregulation of miR-27b was due to decreased transcription of the host gene.

Next, miR-21 caught our attention as one of the most abundantly expressed and studied miRNAs in breast and other cancers^31,32^. RT-qPCR verified low read counts for miR-21. Pri-miR21 levels were also low in MCF7 cells upon HNRNPA1 silencing (Fig. 7B). MiR-21 gene resides within the intron 11 of *VMP1* (Vacuole Membrane Protein-1) on 17q23.2. Hence, we tested whether *VMP1* was also downregulated in HNRNPA1 silenced cells to explain the mechanism behind decreased pri-miR-21 levels. However, there was only a minimal decrease in *VMP1* mRNA, which was unlikely to explain low levels of pri-miR-21 in HNRNPA1 silenced cells (Fig. 7B). However, *VMP1* mRNA is not the only source for miR-21 biogenesis; additional miR-21 promoters and primary transcripts have been characterized from within the terminal intronic regions of *VMP1*^33^. To test whether the activity of this promoter region^34^ was different in HNRNPA1 silenced cells, we cloned the well-defined promoter region for miR-21, a 433 bp region between − 3770 to − 3337 relative to the hairpin, into the pGL3-Basic promoter vector, driving *Firefly* luciferase expression. Transfection efficiency was monitored with phRL-TK driving the expression of the *Renilla* luciferase. Control cells (NT-sh) and HNRNPA1 silenced cells were transiently transfected with both vectors. We observed that the luciferase enzyme activity from the pGL3-miR-21 promoter was approximately 70% lower in HNRNPA1 silenced cells than in the control cells (NT) (Fig. 7C). These findings collectively show that miR-21 and pri-miR-21 levels were downregulated mainly due to the decreased activity of the miR-21 promoter in HNRNPA1 silenced cells.

To test the functionality of miR-21 downregulation on potential targets, we chose to generate a miR-21 sensor rather than testing known mRNA targets because HNRNPA1 loss is likely to alter levels/functions of many other coding and non-coding genes. Hence, we cloned two complementary binding sites for miR-21 downstream of *Firefly* luciferase CDS. We transfected control and HNRNPA1 silenced cells with this sensor. As a result, we detected higher luciferase activity from the miR-21 sensor in *HNRNPA1* silenced cells due to less miR-21 binding to the 3’UTR of the luciferase mRNA (Fig. 7D). A mutant construct lacking the seed sequences of miR-21 had similar luciferase activities in control and HNRNPA1 silenced cells, showing the specificity of the sensor (Supplementary Fig. S10). miR-21 is upregulated in breast cancers, and this upregulation impacts the overall survival of ER+ breast cancers (Supplementary Fig. 10, Fig. 7E). While the effect of HNRNPA1 on miR-21 transcription is possibly indirect, an HNRNPA1 guided network may hold the potential to decipher transcriptional deregulation of miRNAs implicated in cancers.

Finally, we also took an independent approach and targeted the HNRNPA1 gene locus with CRISPR/Cas9. We confirmed decreased expression levels of miRNAs in HNRNPA1 deleted cells (Supplementary Fig. S11); however, the cells were not viable for continued culturing, showing HNRNPA1 dependency of cells. Indeed, most breast cancer cell lines have low “gene effect scores” indicating a high likelihood that HNRNPA1 is an essential gene in depletion assays (Supplementary Fig. S12). Our data and dependency scores collectively suggest that the HNRNPA1 function is critical and that cells cannot rescue its loss. Notably, because disease mutations have been reported for HNRNPA1 along with mutations in HNRNPA2B1^35^, we still looked into expression patterns of the two transcripts. We found no significant correlation in more than a thousand breast cancer patient samples (Supplementary Fig. S12).

Overall, we report an isoform switch for HNRNPA1 and provide insight into the oncogenic roles of HNRNPA1 as a versatile RNA-binding protein whose expression is critical for the neoplastic phenotypes of breast cancer cells. Our findings, specifically on miR-21, may help understand how oncogenic miRNAs are frequently elevated in cancers.

## Discussion

Mechanisms leading to alternative processing of mRNAs are gaining more attention as we begin to appreciate the complexities of cancer transcriptomes^36,37^. Accordingly, widespread expression of alternatively spliced or polyadenylated isoforms has been described in cancers^9,14,38,39^. As part of this complexity, cancer-specific isoform switches change the ratio of mRNA isoforms that may differ in their CDSs or 3’UTR sequences, consequently modulating protein functions in cancer cells. Hence, an increased appreciation of isoform switches may help the discovery of overlooked cancer-related genes and provide new avenues for diagnostic and therapeutic applications.

HNRNPA1 is a versatile protein involved in diverse aspects of RNA biology, including mRNA trafficking, telomere maintenance, regulation of mRNA stability, and splicing by antagonizing or enhancing other splicing proteins. HNRNPA1 can also bind to AU-rich elements and UAGGGA(U)-motifs in the 3’UTRs, and possesses RNA chaperone activity, promoting RNA–RNA interactions. In addition, HNRNPA1 has been implicated in transcriptional activation by binding to and destabilizing G-quadruplex structures within promoters^40–42^. Hence deregulation of HNRNPA1 abundance may have diverse and indirect consequences.

Our work here demonstrates an isoform switch for HNRNPA1 in breast cancers. HNRNPA1 has four similar mRNA isoforms in normal breast tissue as described in the GTEx database. All isoforms mainly differ at their 3′UTRs. Our integrated in silico approach combining isoform level analysis of microarrays, RNA-seq, and single-cell RNA-seq data allowed the discovery and confirmation of the isoform switch. The microarray data clearly showed downregulation of Isoform-1 because distal probes only recognize this isoform. Interestingly, the ratio of other isoforms was high, and the increased expression of these isoform(s) correlated with patient survival. However, it was unclear which isoform was increased because the probes recognized more than one isoform in the microarray data. To this end, the use of GTEx and TCGA datasets revealed isoform-specific expression patterns in breast cancers. Isoform-1, the dominant transcript in mammary tissue, was downregulated in all PAM50 groups compared to GTEx normal tissues. Other minor isoforms (Isoform-3 and 4) were also lower in tumors than adjacent normal or GTEx normal tissue. In contrast, Isoform-2 was the only isoform that was upregulated in breast cancers. This pattern suggested proximal polyadenylation to favor Isoform-2, which has the most proximal poly(A) site, over Isoform-1 and other isoforms with distal poly(A) sites. We wanted to understand the consequence of this switch, and we found that the dominant isoform (Isoform-1) in breast tissue has a unique 3’UTR sequence and is quite unstable, possibly controlling the levels of HNRNPA1 protein. On the other hand, Isoform-2 is stable and translated more than Isoform-1, as was shown by forced expression and reporter assays. These results suggested that this switch may lead to overexpression of HNRNPA1 protein. Indeed independent proteome datasets revealed overexpression of HNRNPA1 protein in tumors and a correlation with poor survival. In support of an oncogenic role, depletion of HNRNPA1 had a significant effect on neoplastic phenotypes in RNAi silenced cell models. The decreased neoplastic phenotypes in vitro were substantial in the RNAi models. Of note, our CRISPR/Cas9 knockout models did not survive. This observation is in agreement with cell dependency scores listed in DepMap and canSAR datasets. Hence these data suggested HNRNPA1 function is critical and possibly not recovered by other members of the HNRNPs.

Given all the diverse roles of HNRNPA1, we sought to provide additional insight into the HNRNPA1 function in breast cancers. A high throughput miRNA expression assay showed a global downregulation of mature miRNA levels. Further analyses showed that the majority of these miRNAs were located within host genes. Among these, we showed miR-21 promoter activity was decreased, and pri-miR-21 levels were downregulated. While the effect on global downregulation of miRNAs is possibly an indirect consequence of HNRNPA1 loss, it will be essential to delineate the HNRNPA1 downstream players responsible for the transcription of pri-miRNAs listed here. Considering these results and the known roles of HNRNPA1 in RNA metabolism^41^, upregulation of HNRNPA1 through the isoform switch may significantly affect different transcriptome components. Of note, while Isoform-2 is upregulated, other isoforms still contribute to HNRNPA1 protein synthesis. The switch enhances protein overexpression but hinders detection of overexpression at the transcript level.

Overall, our data emphasize that focusing on isoform level changes is essential to decoding the cancer transcriptome in higher resolution. This perspective may allow the identification of new oncogene activation cases where overall mRNA levels may not change significantly or common driver mutations do not exist at the genome level. In addition, isoform-specific expression data could also be critical to study isoform-specific post-translational modifications of proteins. Therefore, looking for isoform switches in cancer transcriptomes is a promising strategy to discover new cancer genes with biological impact. The isoform switch we describe for HNRNPA1 has implications in breast cancer and possibly other malignancies.

## Methods

### Isoform level analysis

CEL files of GSE31519, GSE2034, GSE7390 datasets (and normal breast tissue arrays listed in Supplementary Table S4) were analyzed by APADetect for isoform level quantification, as was described^16,17^. Briefly, data sets were analyzed for probe-level differences based on the positions of poly(A) sites. Ratios of proximal to distal probe sets were calculated in normal and cancer samples. Significant changes in the ratio of proximal/distal probe sets, separated by poly(A) sites, were reported as ln(SLR) ((Short + Long)/Long ratio).

### Cell lines

MCF7, MDA-MB-231, and MCF10A cell lines (ATCC HTB-22, HTB-26, and CRL-10317) were grown as suggested by the manufacturer. Cells were checked regularly for mycoplasma contamination by PCR.

### RT-qPCRs and 3′RACE

MIQE guidelines were followed for RT-qPCRs^43^. RNA isolation, cDNA synthesis and RT-qPCR were performed as described^16,17^ (Supplementary Table S1). RPLP0 was used as reference gene for RT-qPRs. Breast cancer cell lines and Breast Cancer cDNA array IV (Origene, BCRT104) were used as described^16,17^. Rapid amplification of cDNA ends (RACE)-specific cDNA synthesis was performed using the 3′ RACE Kit (Roche). Cells were treated with actinomycin D (Tocris Bioscience) (2 µg/mL for MCF7, 10 µg/mL for MDA-MB-231) to determine decay rates. For miRNA quantification, cDNAs were synthesized from total RNA with TaqMan MicroRNA Reverse Transcription Kit (Applied Biosystems, 4427975). TaqMan Universal Master Mix II (Applied Biosystems, 4440040) was used with hsa-miR-21 (000397), hsa-miR-27b (000409), and control RNU43 (001095).

### HNRNPA1 expression and silencing experiments

HNRNPA1 isoforms with Hemagglutinin (HA) tag sequence were PCR amplified (Supplementary Table S2) using Phusion High-Fidelity DNA Polymerase (NEB) and cloned into pcDNA 3.1 (–) (Thermo Fisher). *HNRNPA1* short-hairpin^44^ and non-targeting (NT) shRNA oligos were cloned into pSUPER retro.neo-GFP (OligoEngine) (a gift from Dr. Uygar Tazebay). Two monoclones were picked and expanded. Protein levels were detected by western blotting using anti-HA (Abcam ab9110), anti-HNRNPA1 (Abcam ab177152), and anti-β-actin (Santa-Cruz sc-47778). Bands were visualized in the ChemiDoc MP Imaging System (Bio-Rad). Blots were minimally cut prior to hybridization with HNRNPA1, were stripped off, and re-hybridized with ACTB. Therefore, the whole-length blots are not provided. The raw images for the blots are given in Supplementary Fig. S13.

### Luciferase assays

3′UTRs of isoforms (211, 350 bp, and 702 bp) were cloned into pMIR-Report (pMIR) (Ambion) (Supplementary Table S3). Cells were co-transfected with pMIR (*Firefly* luciferase) and phRL-TK (*Renilla* luciferase) using TurboFect (Thermo Fisher). Twenty-four hours after transfection, cells were collected, and dual luciferase activities were measured with Dual-Luciferase® Reporter Assay (Promega). For promoter activity assay, miR-21 canonical promoter^34^ was cloned into pGL3-Basic and was co-transfected with phRL-TK. For the miR21 sensor, two complementary binding sites were cloned downstream of the luciferase gene into pMIR. Mutant sensors lack the seed sequences of miR21.

### Translating ribosomal affinity purification (TRAP)

EGFP-L10A HEK293 cell line was generated by stable transfection with the pEGFP-C1/RPL10A construct. TRAP was designed and performed as described^45^.

### Datasets

Data were derived from public domain resources. RSEM TPM data in the Genotype-Tissue Expression Project (GTEx) (https://gtexportal.org)^7^ and The Cancer Genome Atlas (TCGA), Genomic Data Commons Data Portal (GDC Data Portal) (https://portal.gdc.cancer.gov) were retrieved from TCGA TARGET GTEx study of UCSC Xena, Xena Toil RNA-Seq Recompute Compendium (https://toil.xenahubs.net) (Jan.16, 2021). TCGA TARGET GTEx study in USCS Xena contains re-analyzed data by the same RNA-Seq pipeline for TCGA and GTEx samples. Thus, the batch effect caused by different computational analyses is eliminated for comparison of tumor vs. normal expression^19^. The clinical data for TCGA-BRCA samples containing PAM50 status were downloaded from TCGA by the TCGAbiolinks R package version 2.20.0^46^. Isoform fraction values were calculated for the four transcripts of *HNRNPA1*. Isoform fractions were calculated by dividing the individual isoform expressions (TPM) by the total expression of all isoforms, as described previously^9^. HNRNPA1 protein levels in tumors were retrieved from Tang et al.^28^. Protein expression for HNRNPA1 in breast cancer subtypes was determined using the CPTAC data from the UALCAN (http://ualcan.path.uab.edu/analysis.html) database^47^. For single-cell RNA-seq data, representative samples were selected from GSE75688 and GSE113197 (Supplementary Table S4). RNA-Seq data analyses were performed through the Cancer Genomics Cloud (CGC), powered by Seven Bridges^48^. Cell dependency scores were retrieved from DepMap portal (https://depmap.org/portal/) using canSAR (https://cansarblack.icr.ac.uk/)^49–53^. Microarray datasets are also listed in Supplementary Table S4.

### Phenotype assays

Colonies were grown for 7 or 14 fourteen days for MCF7 and MDA-MB-231, respectively. Colonies were photographed and counted via the ImageJ^54^. Wounded cell layers were monitored for up to 48 h for wound healing. Images were captured (Olympus Corp.), and wound widths were measured (ImageJ). Colonies were counted and analyzed using CountPHICS (http://www.fuw.edu.pl/~bbrzozow/FizMed/countPHICS.html)^55^. Images of wounds are given in Supplementary Fig. S13. MTT was performed as described^56^. Migration and invasion assays were performed using a transwell system with an 8-µm pore size (Corning). Cells were allowed to migrate for 15 h or invade Matrigel-coated membranes for 18 h. Cells on the apical surface were fixed, stained, and counted as described^56^. All assays were repeated three independent times with at least three technical replicates.

### NanoString nCounter miRNA assay

NanoString nCounter Human miRNA V3 was performed according to manufacturer’s instructions (NanoString Technologies) at CanSyL/M.E.T.U. Significance was calculated using an unpaired t-test for the three technical replicates. Significant expression changes were listed based on fold changes (< 0.6 and > 1.5) and p-values (p < 0.05). Heatmaps were drawn using GraphPad Prism 8.0.2. Biological pathways affected by miRNAs were determined by using DIANA TOOLS mirPath v.3^57^.

### Survival analysis

Expression of isoforms, determined by the proximal probes of 200016_x_at, was used to group patients in the GSE31519 dataset. Patients were grouped according to top 25% (High, n = 90) and bottom 25% (Low, n = 90) expressers. The survival graph for HNRNPA1 protein was from the cohort described in Tang et al.^28^. Hazard ratio (HR) with 95% confidence intervals and log-rank p-value were calculated using Kaplan–Meier Plotter^58^.

## Supplementary Information

Supplementary Information.

## Data Availability

All data generated or analyzed during this study are included in this submitted article and Supplementary Information.
