# Supplementary material for: Identification of an mRNA isoform switch for HNRNPA1 in breast cancers
Source: Sci Rep. 2021 Dec 27;11:24444. doi: 10.1038/s41598-021-04007-y (PMC8712528; doi:10.1038/s41598-021-04007-y)
Supplement: Supplementary file 1 — Supplementary Information. [file 41598_2021_4007_MOESM1_ESM.docx]

**Supplementary Information**

**Identification of an mRNA isoform switch for HNRNPA1 in breast cancers**

Murat Erdem^1*^, İbrahim Ozgul^1*^, Didem Naz Dioken^1^, Irmak Gurcuoglu^1^, Sezen Guntekin Ergun ^2+^, Rengul Cetin-Atalay^2^, Tolga Can^2,3^, Ayse Elif Erson-Bensan^1,2#^

1. Department of Biological Sciences, Middle East Technical University (METU.) Dumlupinar Blv No: 1 Universiteler Mah. Ankara 06800, Turkey
2. Cancer Systems Biology Laboratory, CanSyL, Graduate School of Informatics, Middle East Technical University, 06800, Ankara, Turkey
3. Department of Computer Engineering, Middle East Technical University (METU.) Dumlupinar Blv No: 1 Universiteler Mah. Ankara 06800, Turkey

* These authors should be considered joint first authors.

^+^ Current address: Department of Medical Biology, Hacettepe University, Ankara, Turkey

# Corresponding Author: Ayse Elif ERSON-BENSAN

Department of Biological Sciences

Middle East Technical University

Dumlupinar Blv. No:1, Universiteler Mah no:1

Cankaya 06800 Turkey

Phone:+903122105043

e-mail: erson@metu.edu.tr

**Supplementary Figures**

**Fig. S1**

CDS

Hs.546261.1.27

1. Isoform-1

Forward Primer: 5’- CAGAAGCTCTGGCCCCTATG-3’

CAGAAGCTCTGGCCCCTATGGCGGTGGAGGCCAATACTTTGCAAAACCACGAAACCAAGGTGGCTATGGCGGTTCCAGCAGCAGCAGTAGCTATGGCAGTGGCAGAAGATTTTAATTAGGGAGGAGTCTGCTACTAGTCTTATCAGCTCTTAAAAACAGAAACTCATCTGTCCAAGTTCGTGGCAGAAAGGAACGTCCTTGTGAAGACCTTTATCTGAGCCACTGTACTTCGTTATCACTGCCATGCAGTTTACATGAGCTGTTCTGCAGCTCAAATTCCATTTTGTGAATGGGTTTTTTTTTTTAATAAACTGTATTTAACTC**AAAAAAAAAAAAAAAA**GTCGACATCGATACGCGTGGTC

RACE Anchor Oligo dT: 5’-GACCACGCGTATCGATGTCGACTTTTTTTTTTTTTTTT-3’


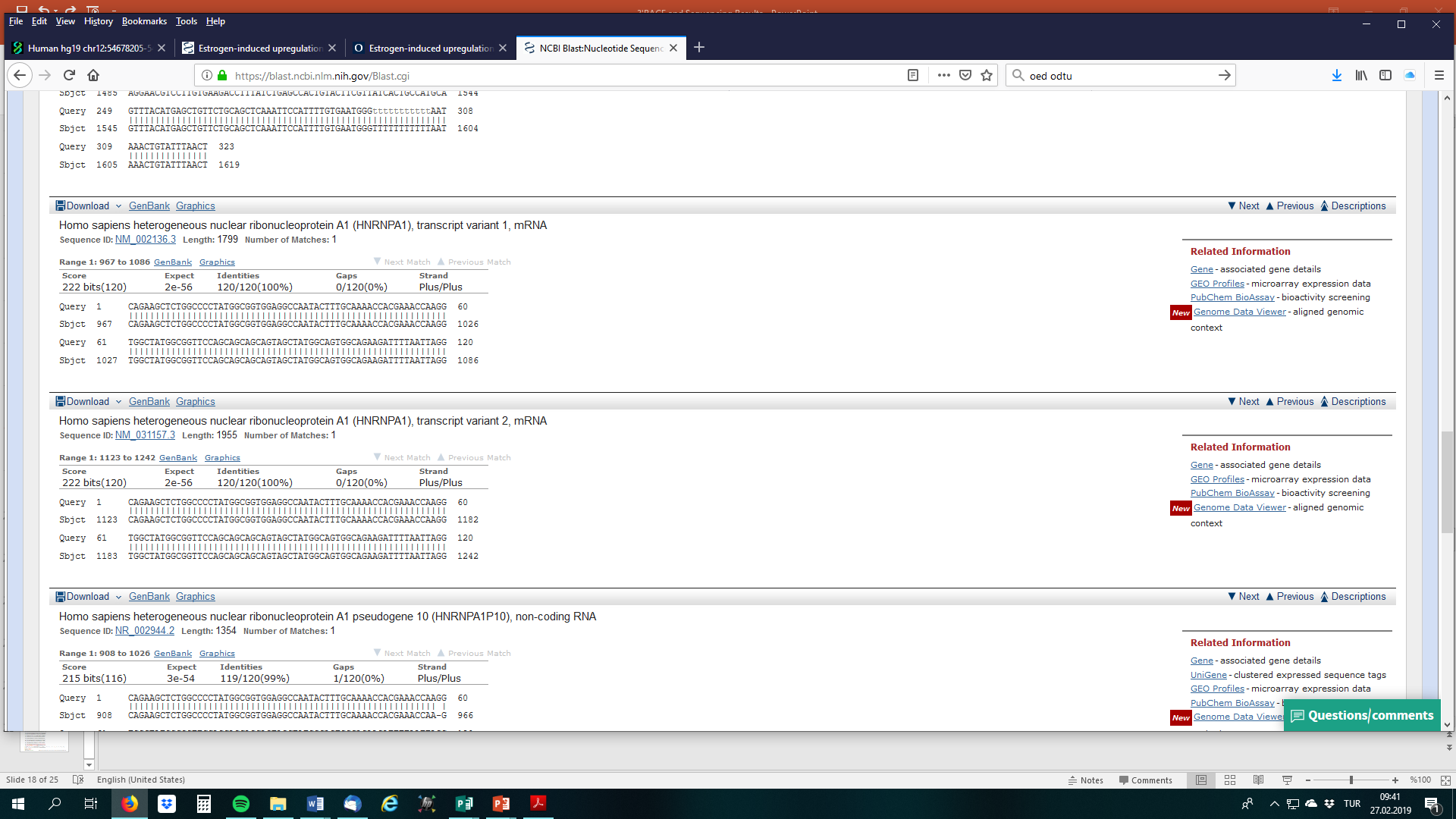


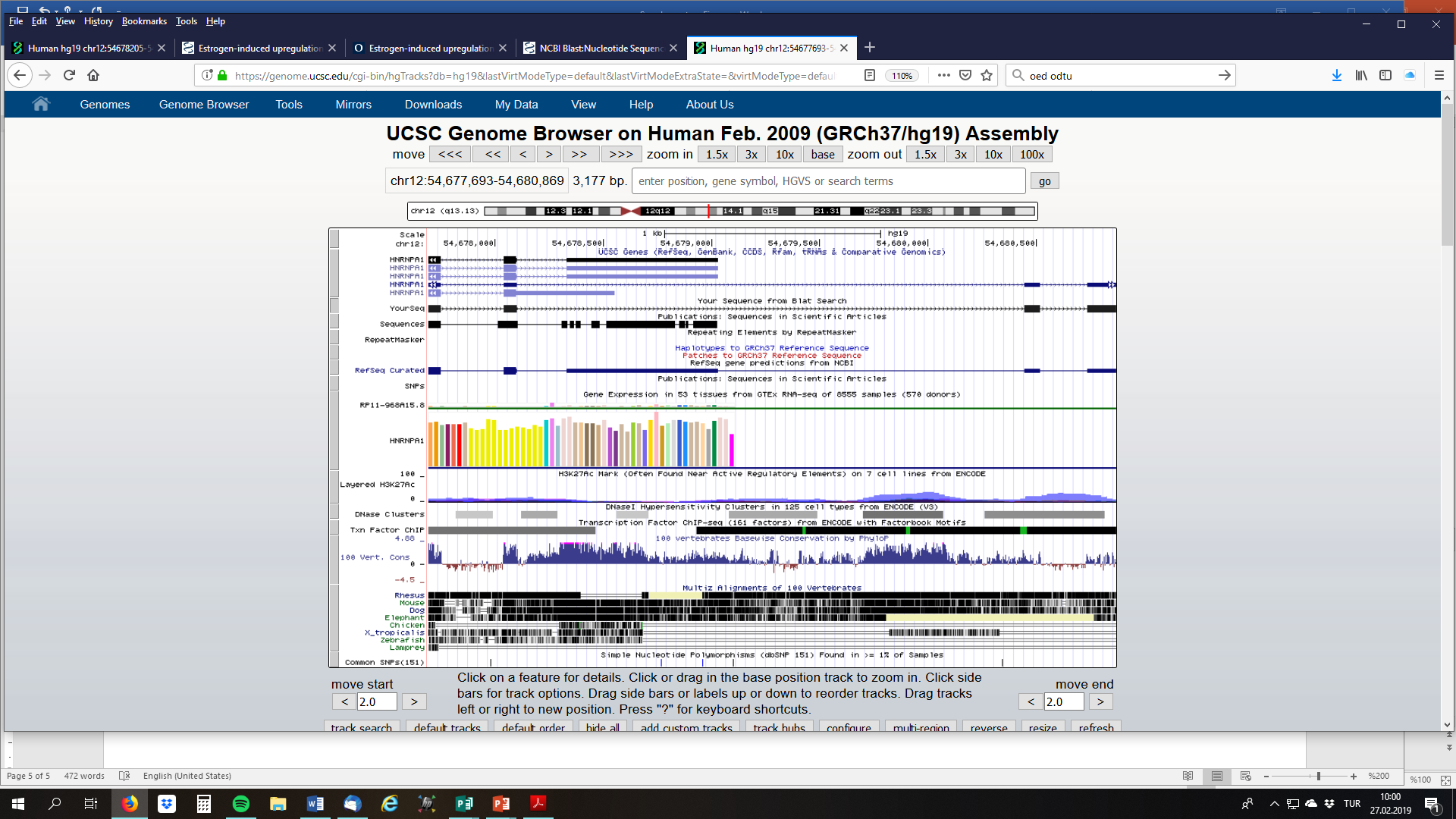


**Supplementary Fig. S1.** Hs.546261.1.27 poly(A) site. **A**. 3’RACE for the transcript that is polyadenylated at Hs. Hs.546261.1.27. Forward and reverse (anchor) primers are underlined on the sequence. The sequenced PCR product confirms the end of the transcript at Hs.546261.1.27. The coding sequence is highlighted in grey. **B.** 3’RACE product was sequenced and blasted against HNRNPA1 (NCBI-BLAST) and analyzed with Blat tool against GRCh37/hg19 genome assembly (UCSC Blat).

**Fig. S2**

Hs.546261.1.20

CDS

1. Short 3’UTR

Forward Primer: 5’-CAGAAGCTCTGGCCCCTATG-3’

CAGAAGCTCTGGCCCCTATGGCGGTGGAGGCCAATACTTTGCAAAACCACGAAACCAAGGTGGCTATGGCGGTTCCAGCAGCAGCAGTAGCTATGGCAGTGGCAGAAGATTTTAATTAGGAAACAAAGCTTAGCAGGAGAGGAGAGCCAGAGAAGTGACAGGGAAGCTACAGGTTACAACAGATTTGTGAACTCAGCCAAGCACAGTGGTGGCAGGGCCTAGCTGCTACAAAGAAGACATGTTTTAGACAAATACTCATGTGTATGGGCAAAAAACTCGAGGACTGTATTTGTGACTAATTGTATAACAGGTTATTTTAGTTTCTGTTCTGTGGAAAGTGTAAAGCATTCCAACAAAGGGTTTTAATGTAGATTTTTTTTTTTGCACCCCATGCTGTTGATTGCTAAATGTAACAGTCTGATCGTGACGCTGAATAAATGTCTTTTTTTTAAAAAAAAAAAAAAAAGTCGACATCGATACGCGTGGTC

RACE Anchor Oligo dT: 5’-GACCACGCGTATCGATGTCGACTTTTTTTTTTTTTTTTV-3’


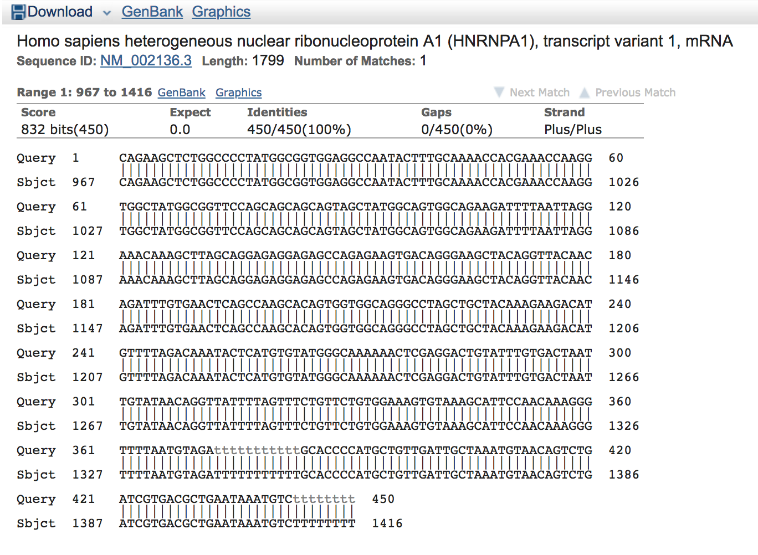


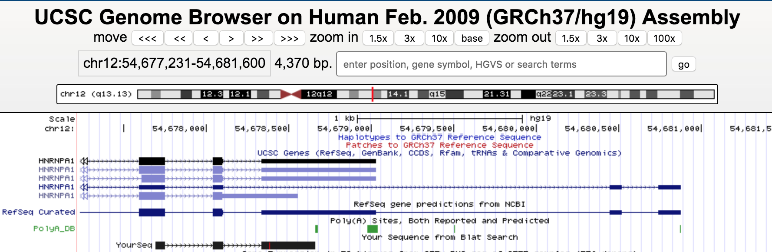


**Supplementary Fig. S2.** Hs.546261.1.20 poly(A) site. **A**. 3’RACE for the transcript that is polyadenylated at Hs.546261.1.20. Forward and reverse (anchor) primers are underlined on the sequence. The sequenced PCR product confirms the end of the transcript at Hs.546261.1.20. The coding sequence is highlighted in grey. **B.** 3’RACE product was sequenced and blasted against HNRNPA1 (NCBI-BLAST) and analyzed with Blat tool against GRCh37/hg19 genome assembly (UCSC-Blat).

**Fig. S3**

Hs.546261.1.20

CDS

Hs.546261.1.23

1. Long 3’UTR

Forward Primer: 5’-CAGAAGCTCTGGCCCCTATG-3’

CAGAAGCTCTGGCCCCTATGGCGGTGGAGGCCAATACTTTGCAAAACCACGAAACCAAGGTGGCTATGGCGGTTCCAGCAGCAGCAGTAGCTATGGCAGTGGCAGAAGATTTTAATTAGGAAACAAAGCTTAGCAGGAGAGGAGAGCCAGAGAAGTGACAGGGAAGCTACAGGTTACAACAGATTTGTGAACTCAGCCAAGCACAGTGGTGGCAGGGCCTAGCTGCTACAAAGAAGACATGTTTTAGACAAATACTCATGTGTATGGGCAAAAAACTCGAGGACTGTATTTGTGACTAATTGTATAACAGGTTATTTTAGTTTCTGTTCTGTGGAAAGTGTAAAGCATTCCAACAAAGGGTTTTAATGTAGATTTTTTTTTTTGCACCCCATGCTGTTGATTGCTAAATGTAACAGTCTGATCGTGACGCTGAATAAATGTCTTTTTTTTAATGTGCTGTGTAAAGTTAGTCTACTCTTAAGCCATCTTGGTAAATTTCCCCAACAGTGTGAAGTTAGAATTCCTTCAGGGTGATGCCAGGTTCTATTTGGAATTTATATACAACCTGCTTGGGTGGAGAAGCCATTGTCTTCGGAAACCTTGGTGTAGTTGAACTGATAGTTACTGTTGTGACCTGAAGTTCACCATTAAAAGGGATTACCCAAGCAAAATCATGGAATGGTTATAAAAGTGATTGTTGGCACATCCTATGCAATATATCTAAATTGAATAATGGTACCAGATAAAATTATAGATGGGAATGAAGCTTGTGTATCCATTATCATGTGTAATCAATAAACGATTTAATTCTCTTGAAAAAAAAAAAAAAAGTCGACATCGATACGCGTGGTC.

RACE Anchor Oligo dT: 5’-GACCACGCGTATCGATGTCGACTTTTTTTTTTTTTTTTV-3’


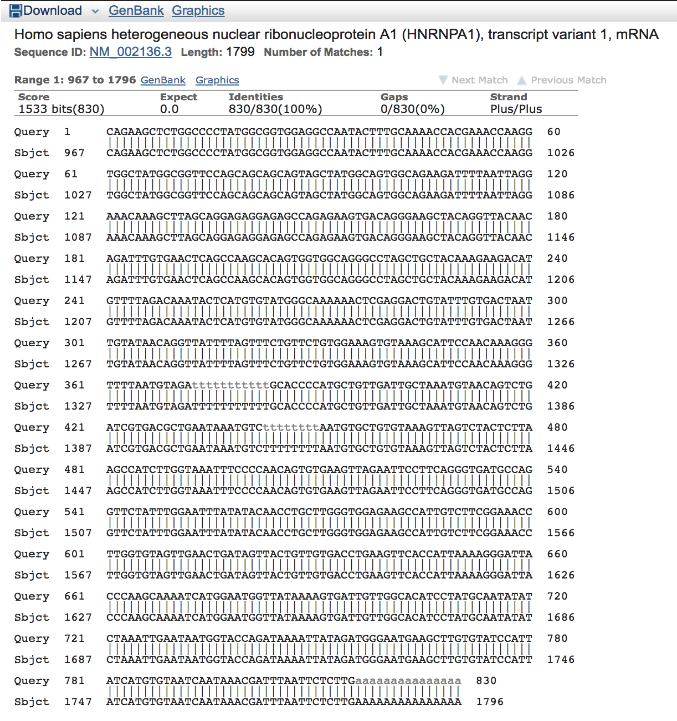


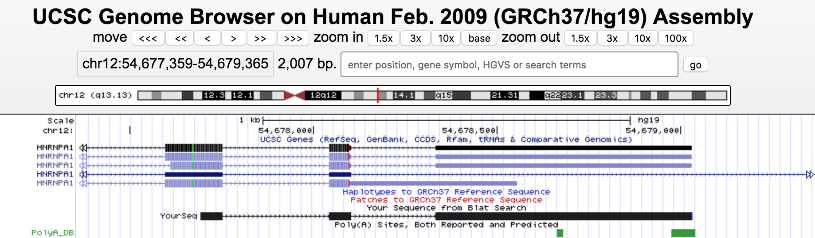


**Supplementary Fig. S3.** Hs.546261.1.23 poly(A) site. **A**. 3’RACE for the transcript that is polyadenylated at Hs.546261.1.23. Forward and reverse (anchor) primers are underlined on the sequence. The sequenced PCR product confirms the end of the transcript at Hs.546261.1.23. The coding sequence is highlighted in grey. **B.** 3’RACE product was sequenced and blasted against HNRNPA1 (NCBI-BLAST) and analyzed with Blat tool against GRCh37/hg19 genome assembly (UCSC-Blat).

**Fig. S4.**

**A.**


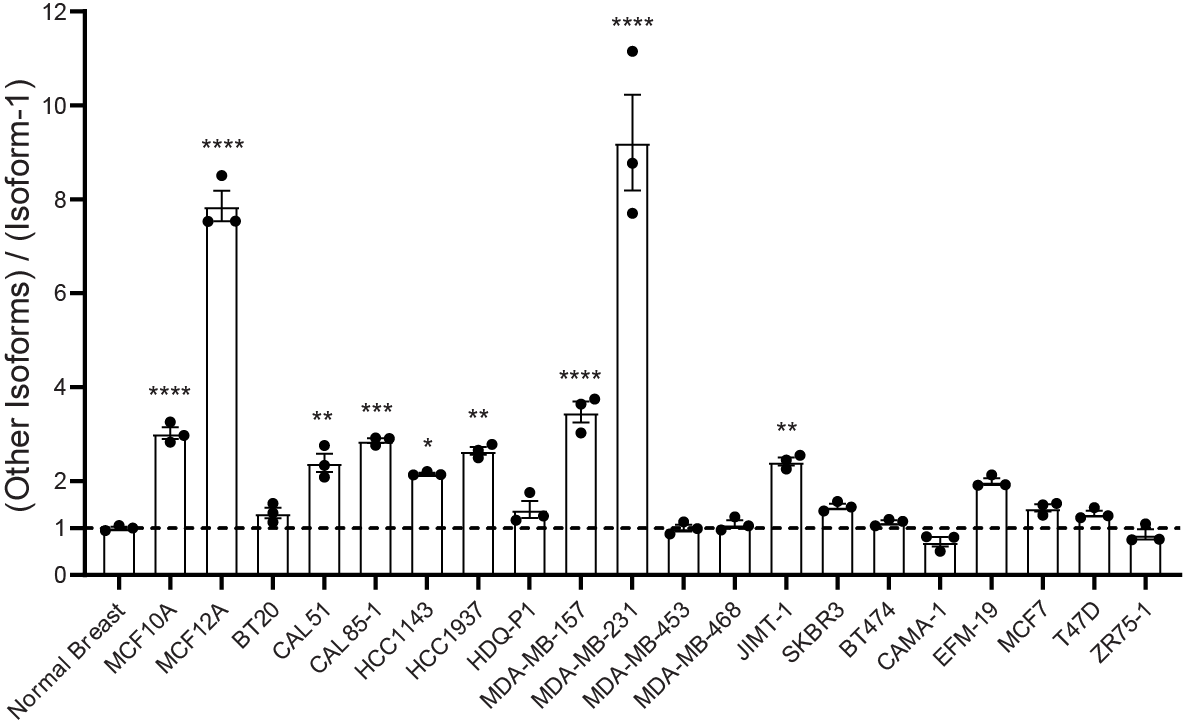


**B.**


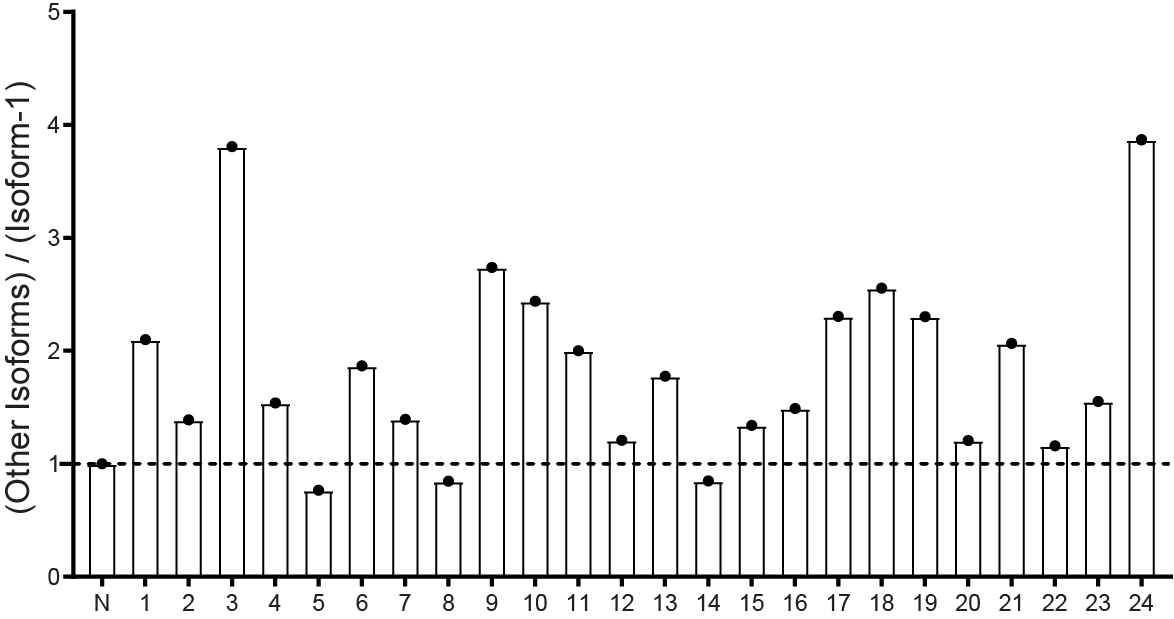


**Supplementary Fig. S4.** Isoform switch in breast cancer cell lines and in patients. **A**. Ratio of *HNRNPA1* isoforms in breast cancer cell lines (BT20, CAL51, CAL85-1, HCC1143, HCC1937, HDQ-P1, MDA-MB-157, MDA-MB-231, MDA-MB-453, MDA-MB-468, JIMT-1, SKBR3, BT474, CAMA-1, EFM-19, MCF7, T47D, ZR75-1) were determined by RT-qPCR. (n=3 technical replicates for each cell line, *p<0.05, **p<0.01, ***p<0.001, ****p<0.0001, two-way ANOVA, Dunnett's multiple comparisons test) **B.** Ratio of *HNRNPA1* isoforms in a panel of breast cancer patient cDNAs (Origene Breast cDNA array IV). *HNRNPA1* Isoform-2, Isoform-3 and Isoform-4 were co-amplified by common primers. Isoform-1 was amplified using specific primers to its unique 3’UTR (n=1 due to limited material). The fold change for the isoforms was normalized against the reference gene, *RPLP0,* and normalized to normal breast tissue RNA (Thermo Fisher Scientific). The ratio was calculated as (Other Isoforms)/(Isoform-1).

**Fig. S5.**

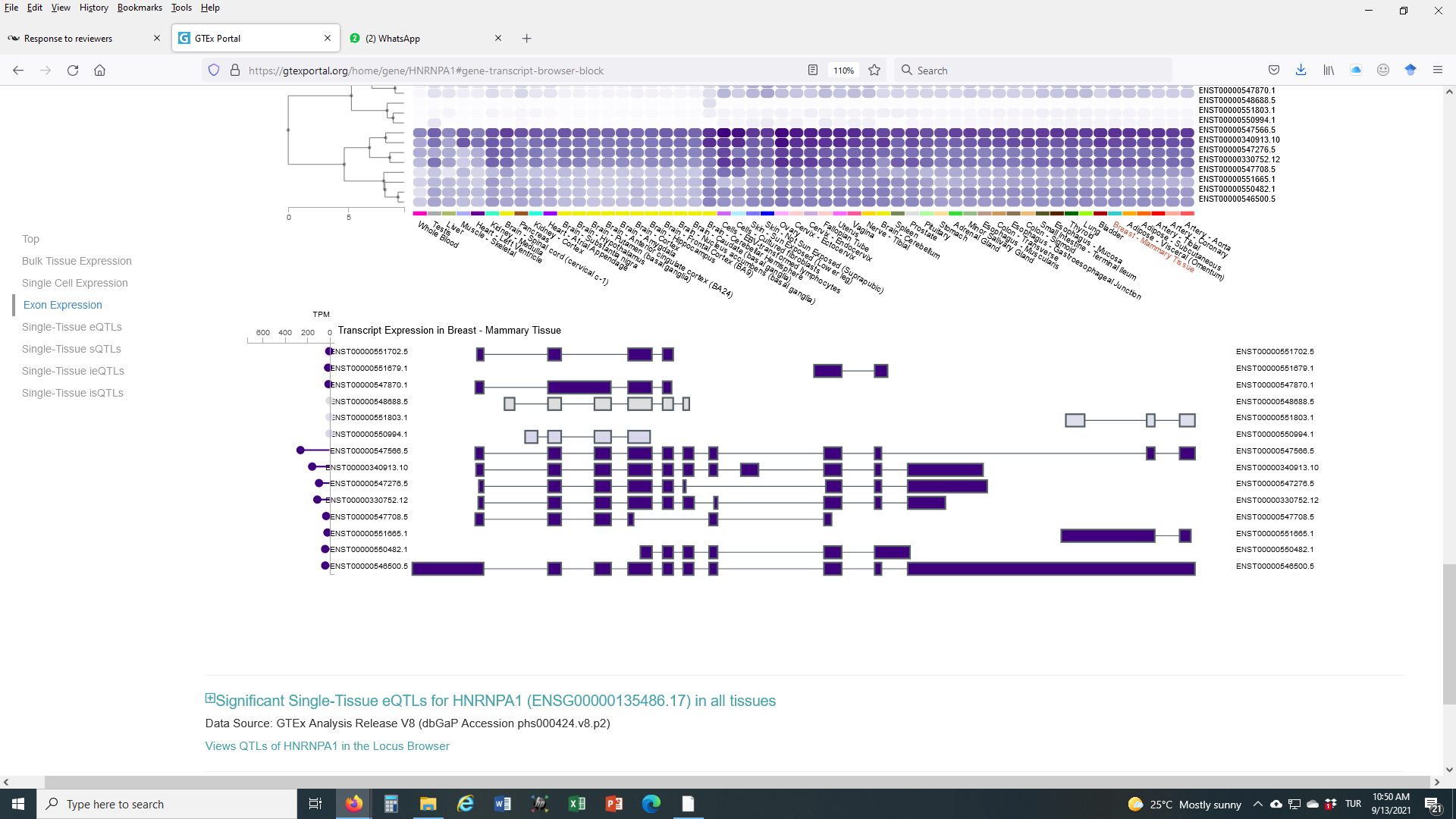


**Supplementary Fig. S5.** *HNRNPA1* gene structure. Triangles indicate polyadenylation sites. CDS starts at exon 1 and ends at exon 10. **B.** HNRNPA1 isoforms in normal mammary tissue in the GTEx database.

**Fig. S6**


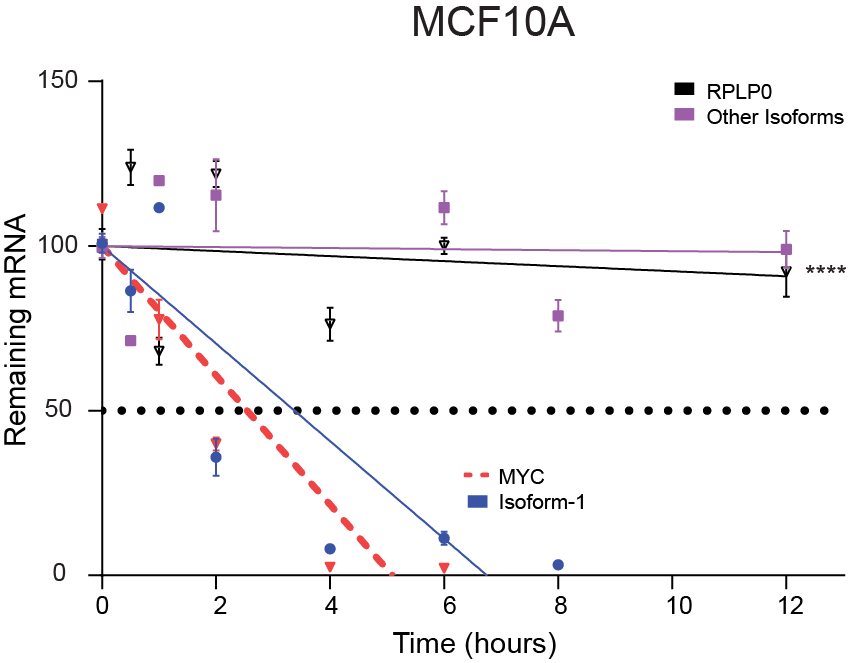


| **mRNAs** | **t(1/2) hours** |
| --- | --- |
| MYC | 2.6 |
| Isoform 1 | 3.4 |

**Supplementary Fig. S6.** Stability of *HNRNPA1* mRNA isoforms in MCF10A (non-tumorigenic mammary epithelial cells). Cells were treated with actinomycin D (10 µg/ml) for indicated time points. RNA was isolated for RT-qPCR. mRNA decay rates (t_1/2_) for Isoform 1 and *MYC* in MCF10A cells are shown in the bottom panel (****p<0.0001, n=2 treatments with three technical replicates, one-way ANOVA, Tukey's HSD).

**Fig. S7**


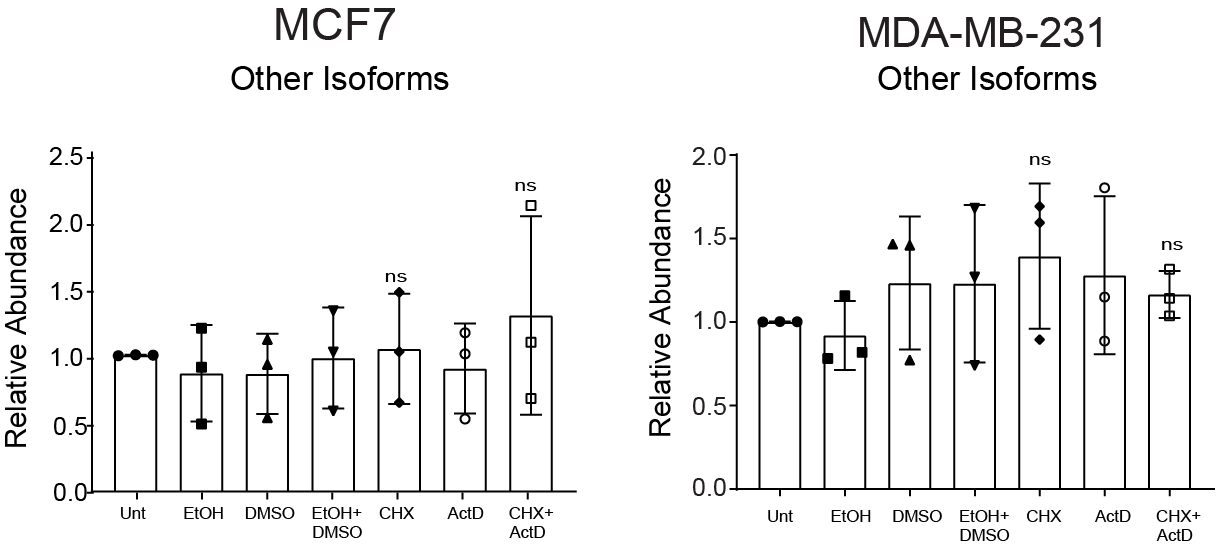


**Supplementary Fig. S7.** Stability of *HNRNPA1* mRNA isoform-2, 3 and 4 in MCF7 and MDA-MB-231 cells. Cells were treated with actinomycin D and/or cycloheximide (CHX, 100 µg/mL) for 3 hours to prevent transcription and translation. EtOH (Ethanol) and DMSO are carrier controls. Cells were collected, and RNA was isolated for RT-qPCR (ns: not significant, n=3 independent experiments, student’s t-test).

**Fig. S8**


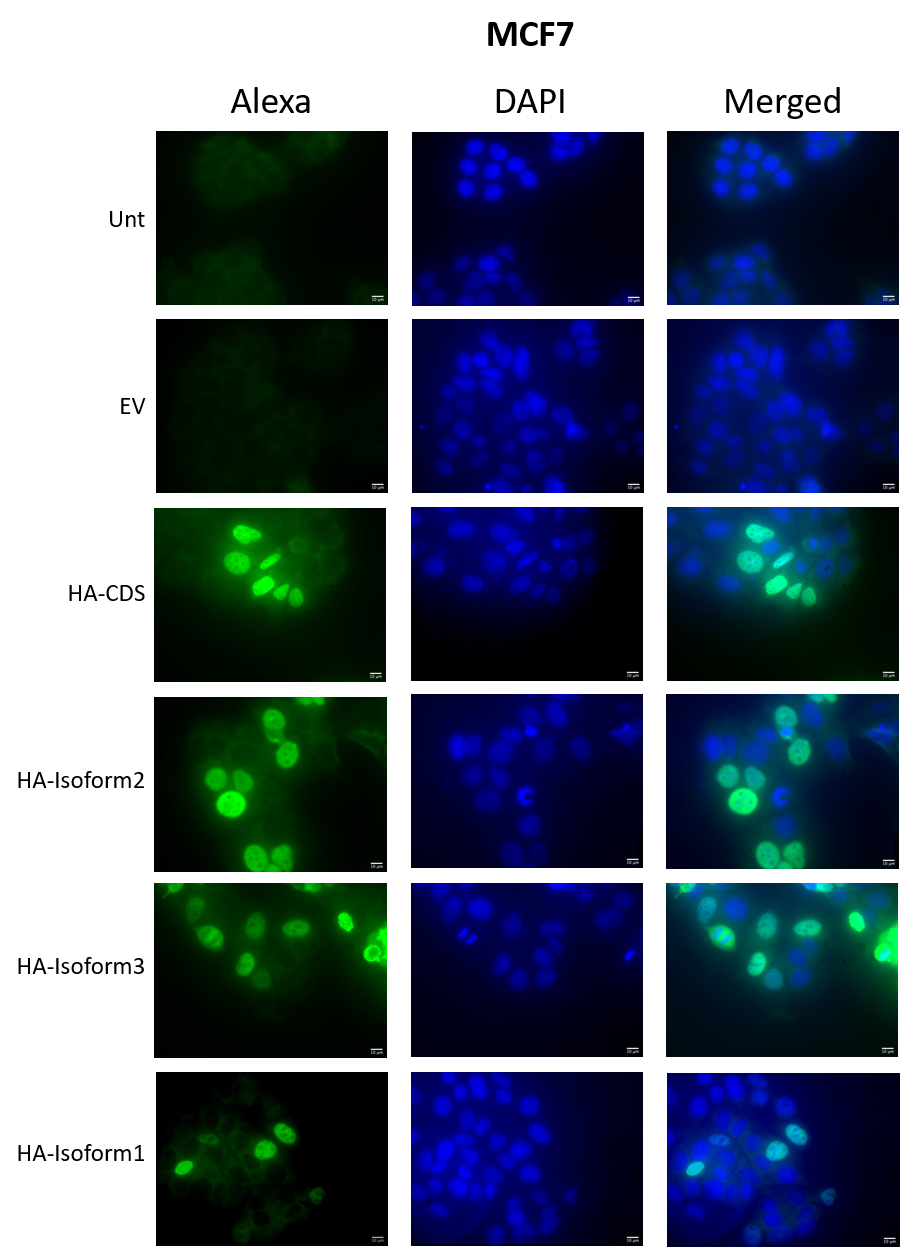


**Supplementary Fig. S8.** Subcellular localization of HNRNPA1 protein translated from isoforms. After 48 hours of transfection, HA-tagged HNRNPA1 translated from Isoform-1, Isoform-2, and Isoform-3 (CDS+3’UTRs) were detected by immunocytochemistry in MCF7 cells. Alexa (HA-HNRNPA1): Green, DAPI: Blue. Scale bars: 10 μm. Unt: Untransfected MCF7 cells, EV: Empty vector (pcDNA 3.1 (-)) transfected MCF7 cells. MCF7 cells were fixed with 2% paraformaldehyde, permeabilized with 0.4% Triton X-100 in PBS, blocked with 10% BSA, and incubated with rabbit anti-HA-tag monoclonal antibody (1:500, Abcam, ab9110, RRID: AB_307019) at RT 1 hour. Cells were stained with the Alexa Fluor 488-conjugated goat polyclonal anti-rabbit IgG secondary antibody (1:1000; Abcam, ab150077, RRID:AB_2630356) and counterstained with fluoroshield mounting medium with DAPI (Abcam, ab104139).

**Fig. S9**


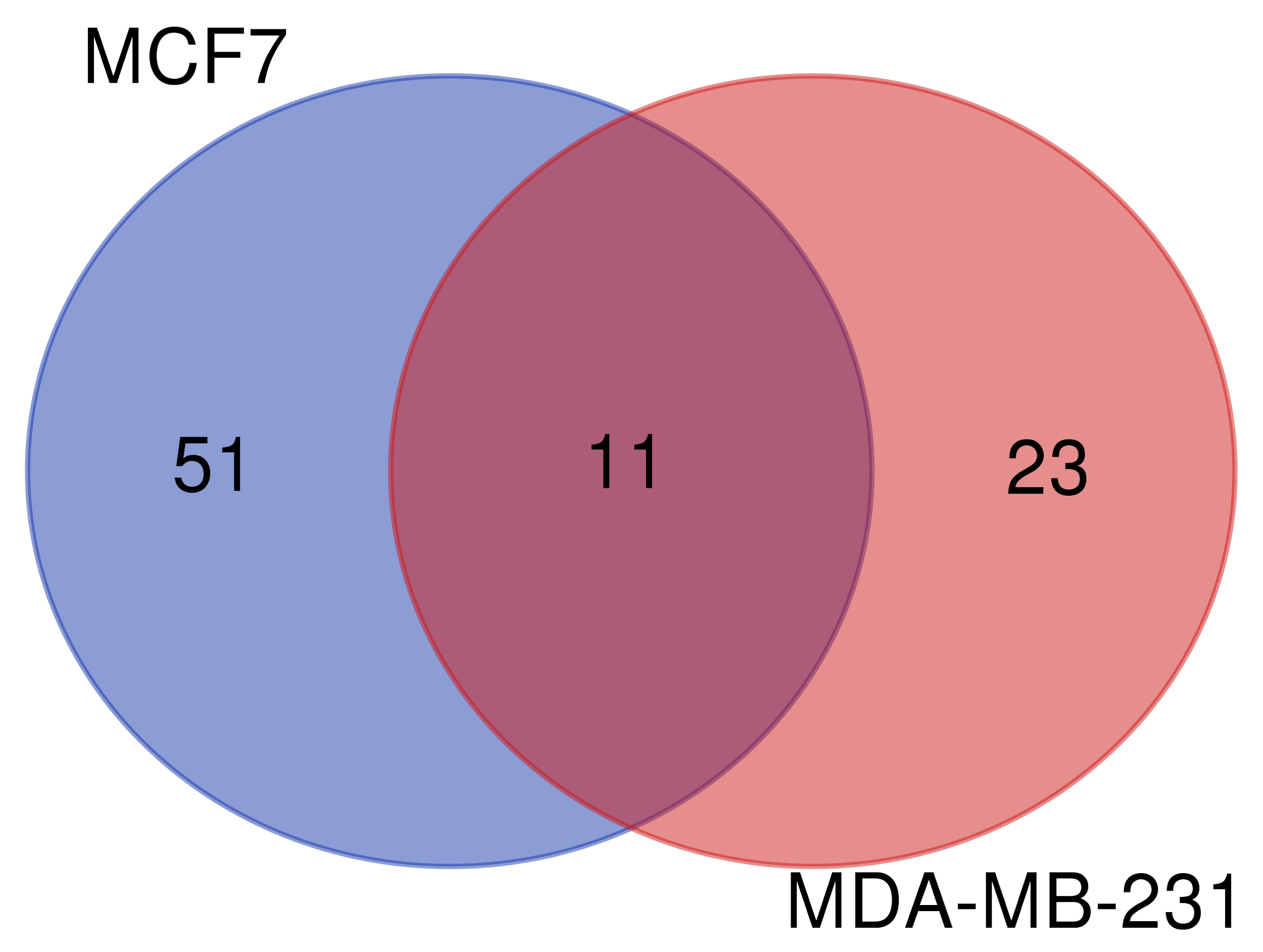


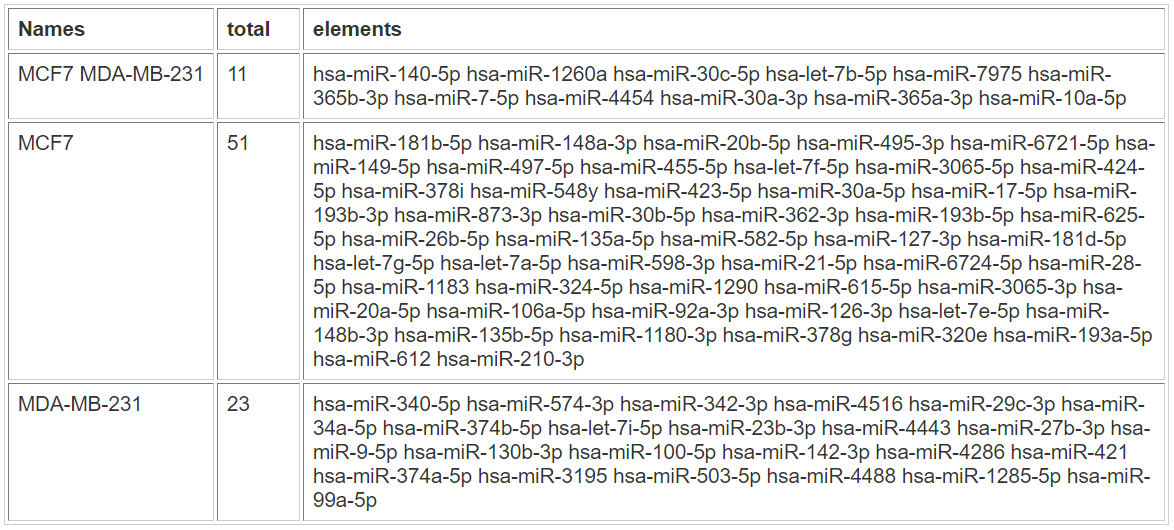


**Supplementary Fig. S9.** miRNA lists **A.**Venn diagram of cell line-specific and /or common miRNAs whose expression is significantly altered upon long-term silencing of HNRNPA1. **B.** The list of common and cell line-specific miRNAs in HNRNPA1 silenced MCF7 and MDA-MB-231 cells.

**Fig. S10**

**
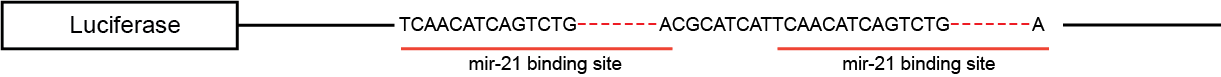
**


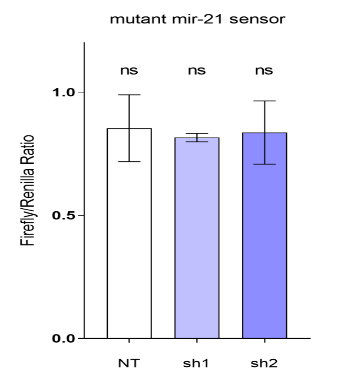


**C.**


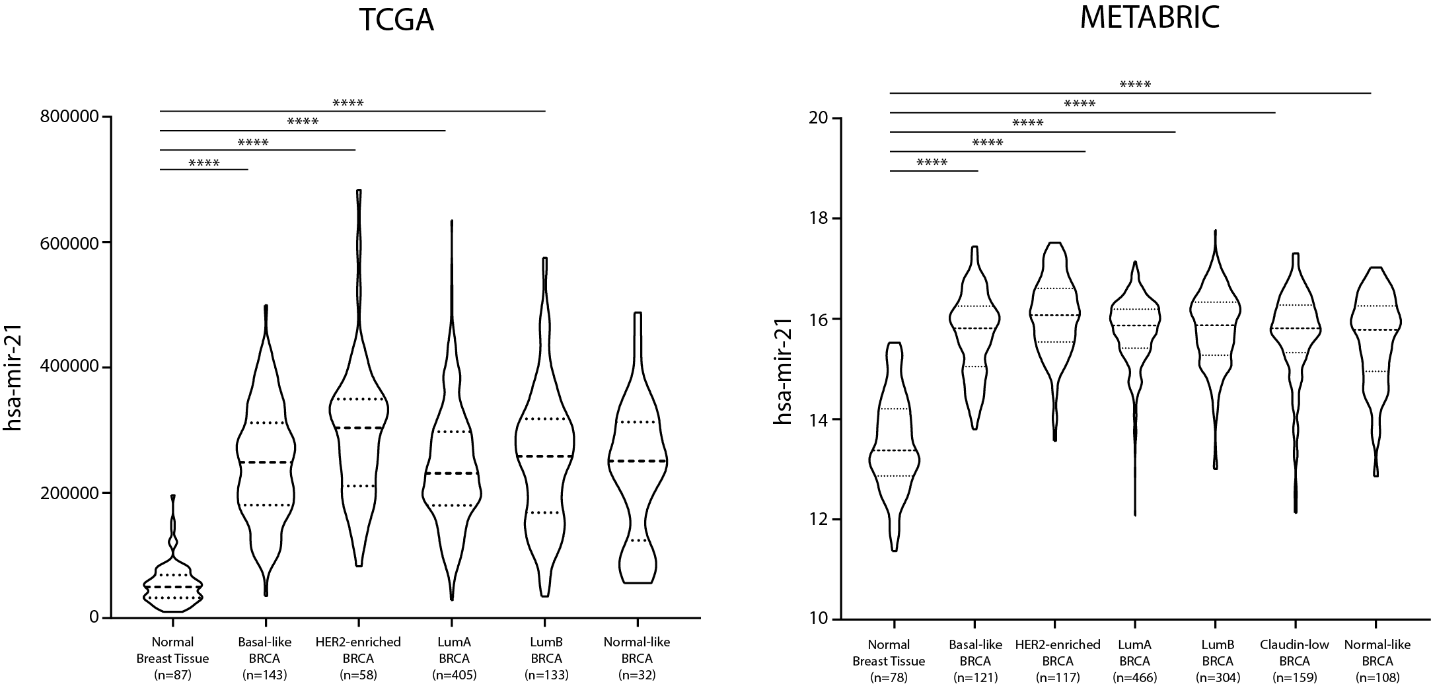


**Supplementary Fig. S10.** Mutant miR21 sensor. **A.** Structure of the mutant miR-21 sensor. Two reverse complementary miR-21 binding sequences without seed sequences, separated by 8-nucleotide spacer were cloned downstream of the *Luciferase* gene. **B.** Mutant miR21 sensor (*Firefly luciferase*) and *Renilla luciferase* vectors were co-transfected to MCF7 cells (NT: Control, non-targeting shRNA, sh1 and sh2 are HNRNPA1 silenced cells). *Firefly/Renilla luciferase* read-outs from the constructs were normalized to that of empty vector (ns: not significant, n=3 independent transfections, one-way ANOVA, Tukey's HSD). **C.** Normalized hsa-mir-21 levels across breast cancer subtypes and normal breast tissue from TCGA miRNA data retrieved from OncoMir Cancer Database (OMCD) (https://www.oncomir.umn.edu/omcd/) (left panel) and METABRIC Data ID: EGAD00010000438 (right panel) (Wong NW et al. 2018), (****p<0.0001, one-way ANOVA, Tukey's HSD).

**Fig. S11**

C

MCF7
C1

MDA-MB-231
C1

MCF7
C2

MDA-MB-231
C2

x y z


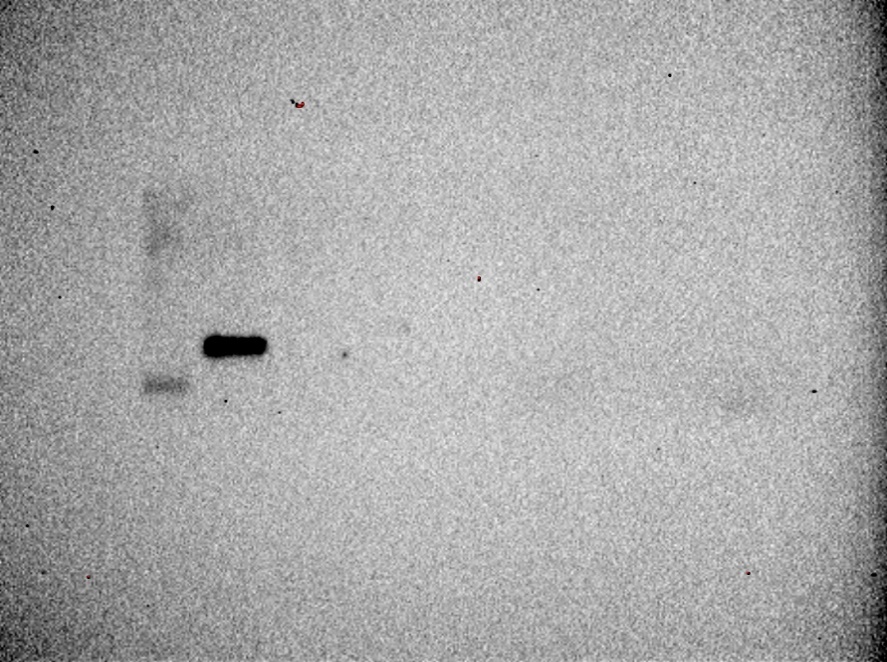


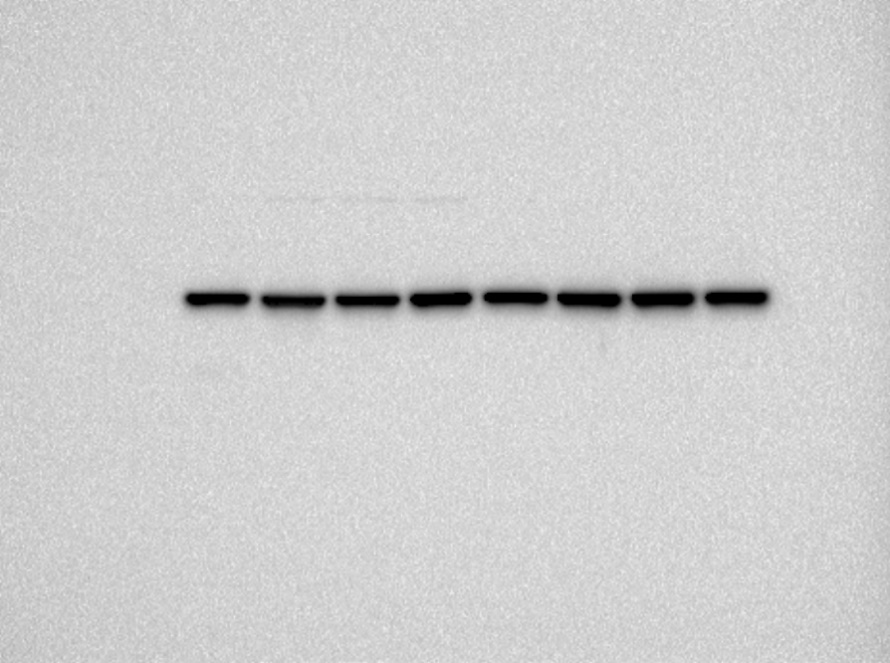


42 kDa

x y z

ACTB

HNRNPA1

34 kDa

C

MCF7
C1

MCF7
C2

MDA-MB-231
C1

MDA-MB-231
C2

**B.**

**
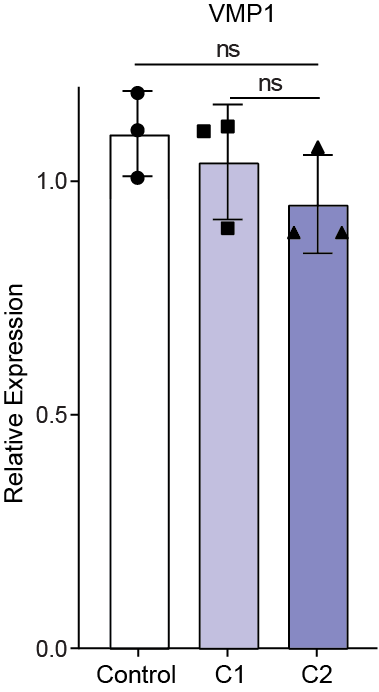

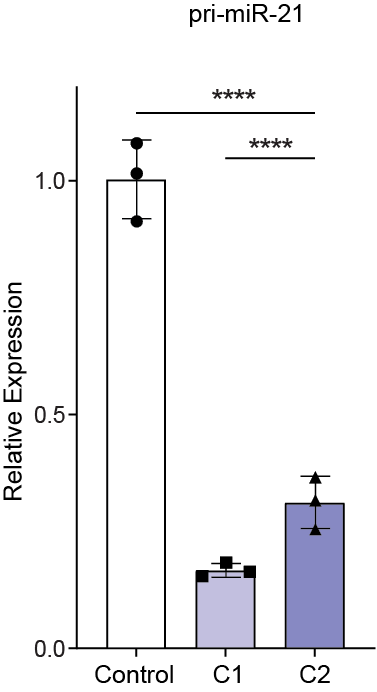

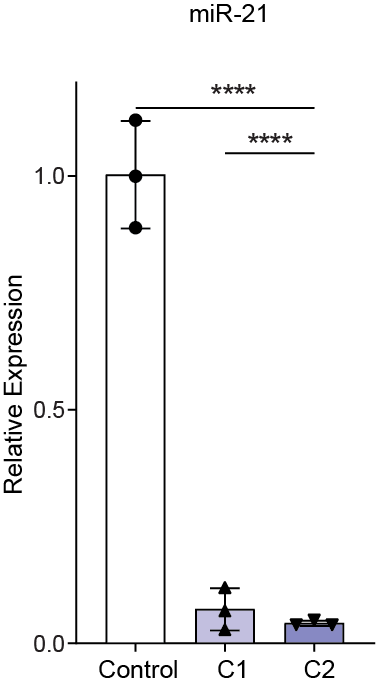
**

**
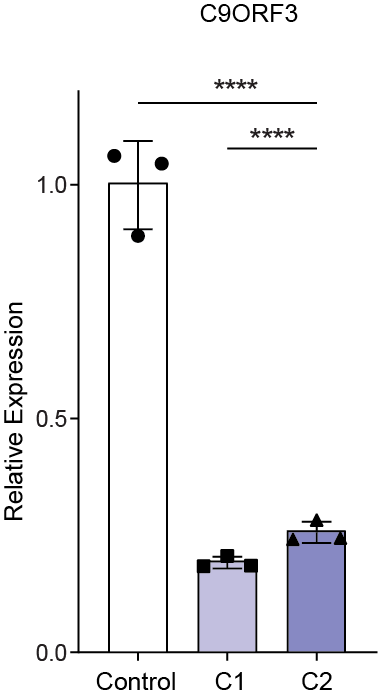

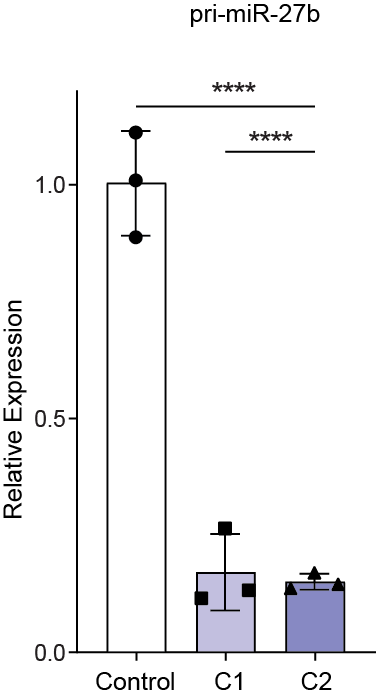
**

**Supplementary Fig. S11.** Genomic deletion of HNRNPA1. **A.** HNRNPA1 was deleted in MCF7 and MDA-MB-231 cells using CRISPR/Cas9. Lysates were collected from the clones for western blotting using to confirm the lack of HNRNPA1 protein. ACTB antibody was used as a loading control on the same blot (n=1). Blue dashed boxes show the selected clones (C1, C2). The lanes outside the box (lane x, y, z) are other CRISPR clones that were not used for further analyses. **B.** Relative expression levels of *VMP1*, pri-miR21, miR21 in MCF7 and C9ORF3, pri-miR-27b levels in MDA-MB-231 cells (HNRNPA1 knockouts). *RNU43* was used as the reference gene for miR-21, and *RPLP0* was used as a reference gene for other RT-qPCRs. C1 and C2 indicate individual colonies. RT-qPCRs were repeated three times (ns: not significant for *VMP1*, ****p<0.0001, One-way ANOVA, Tukey's HSD).

**Fig. S12**


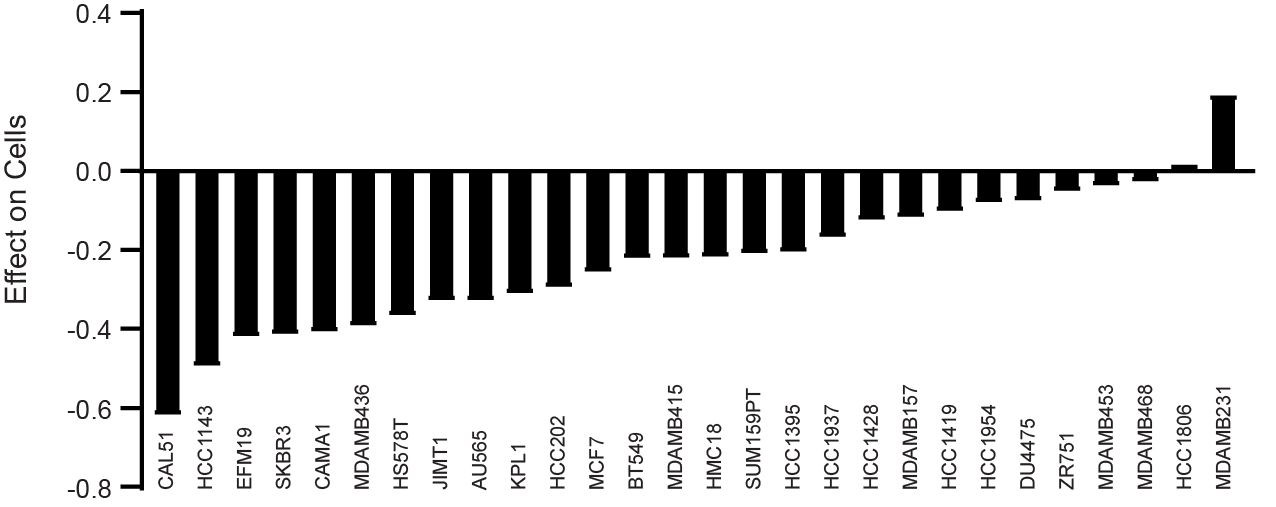


**A.**


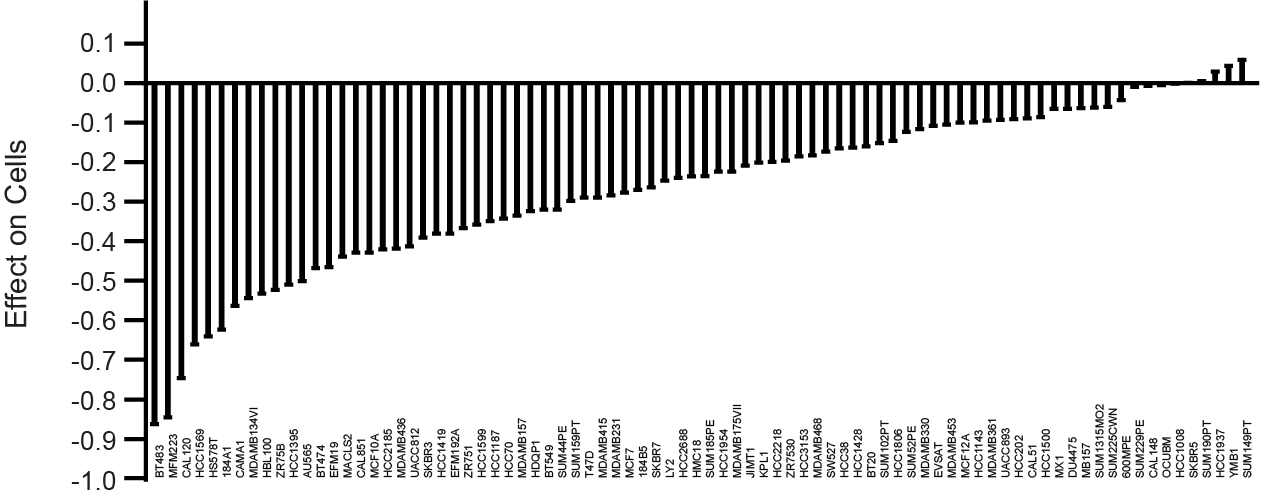


**B.**

**C**.


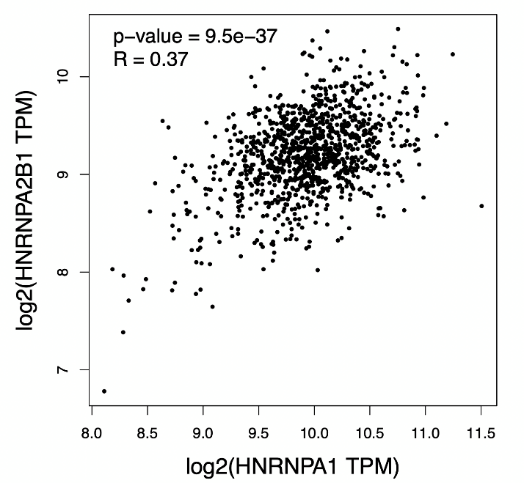


**Supplementary Fig. S12.** **A**. CRISPR dependency scores for HNRNPA1. Gene effect scores express how vital HNRNPA1 is in terms of lethality in breast cancer cell lines. **B.** RNAi dependency scores for HNRNPA1. Scores represent the observed log fold change in the amount of shRNA detected in cancer cell lines. A highly negative dependency score implies that a cell line is highly dependent on HNRNPA1. Data were retrieved from DepMap portal (https://depmap.org/portal/) using canSAR (https://cansarblack.icr.ac.uk/). **C.** Lack of a significant correlation (R=0.37) between the gene expression levels of *HNRNPA1* and *HNRNPA2B1* in TCGA BRCA Dataset (n=1085), determined by the GEPIA2 (http://gepia.cancer-pku.cn/) platform using the Spearman correlation coefficient method.

**Fig. S13.**

Western Blot images for Fig. 3C.

**Replicate 1: MCF7** – HA-HNRNPA1 Western Blot image

Last sample (lane x) was a positive control lysate expressing HA-HNRNPA1.


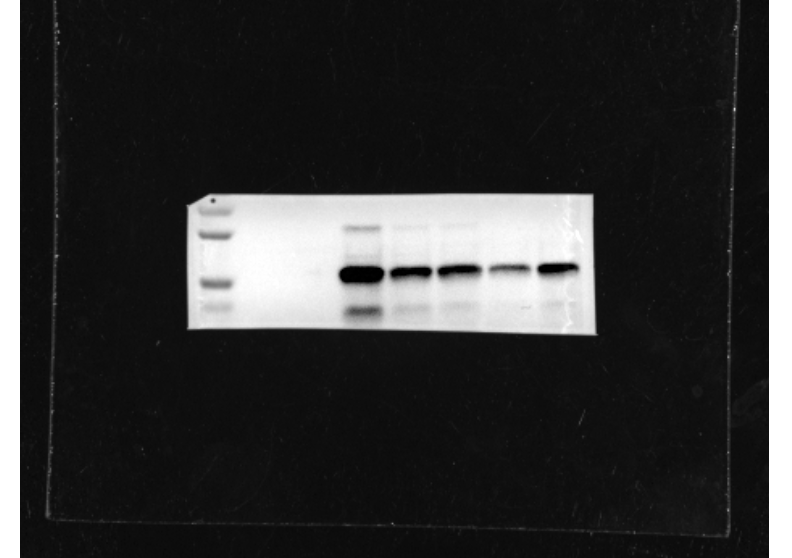


25 kDa

34 kDa

70 kDa

55 kDa

b

c

d

x

C

a

EV

HA-HNRNPA1

MCF7 – HA-HNRNPA1 (longer exposure of the same blot)

x

EV

d

c

b

a

C


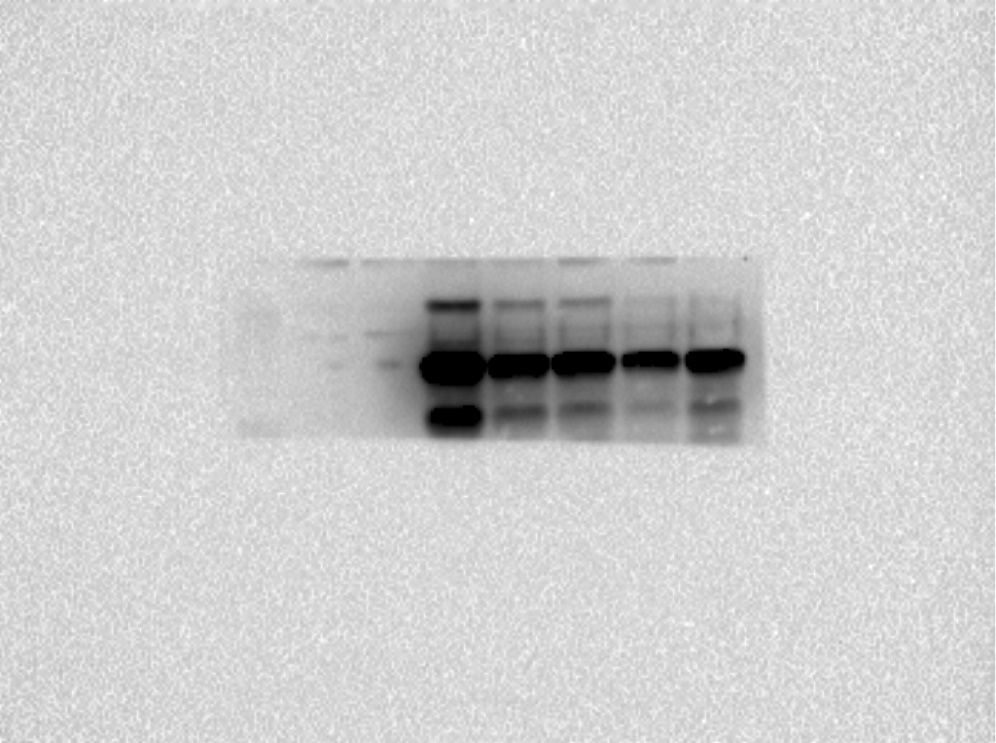


HA-HNRNPA1

MCF7 – ACTB Western Blot image (same blot)

x

EV

C

a

b

c

d


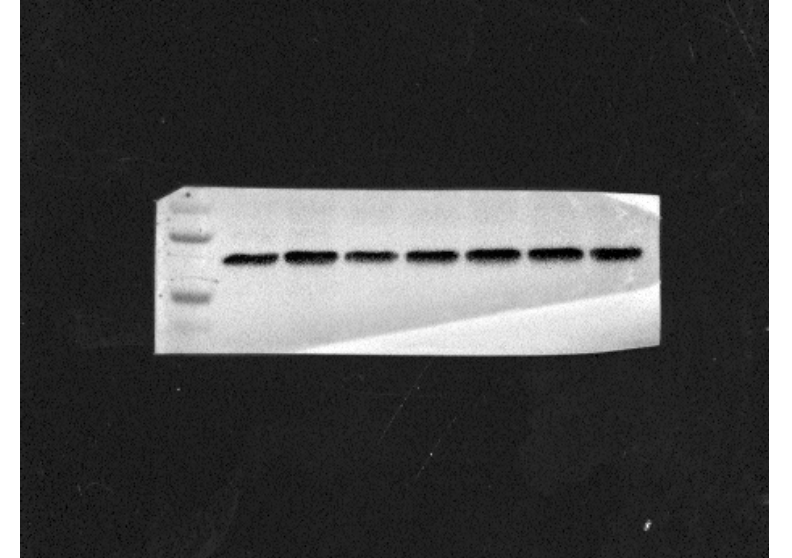


70 kDa

34 kDa

55 kDa

ACTB

**Replicate 2:** Blue dashed boxes show the presented data in the Fig. 3C.

**MCF7**

c

b

d

a

EV

C

**
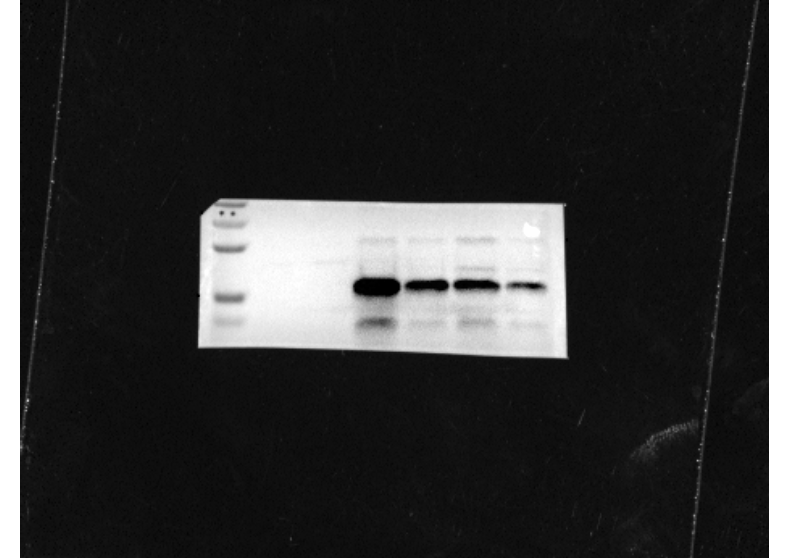
**

70 kDa

55 kDa

HA-HNRNPA1

25 kDa

34 kDa

b

d

c

a

C

EV


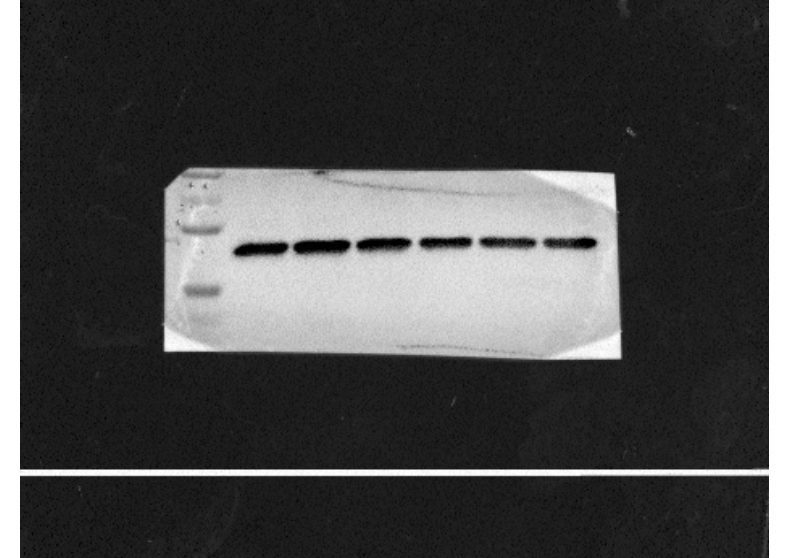


55 kDa

70 kDa

25 kDa

ACTB

34 kDa

**Replicate 3:**

d

c

b

a

EV

C

**
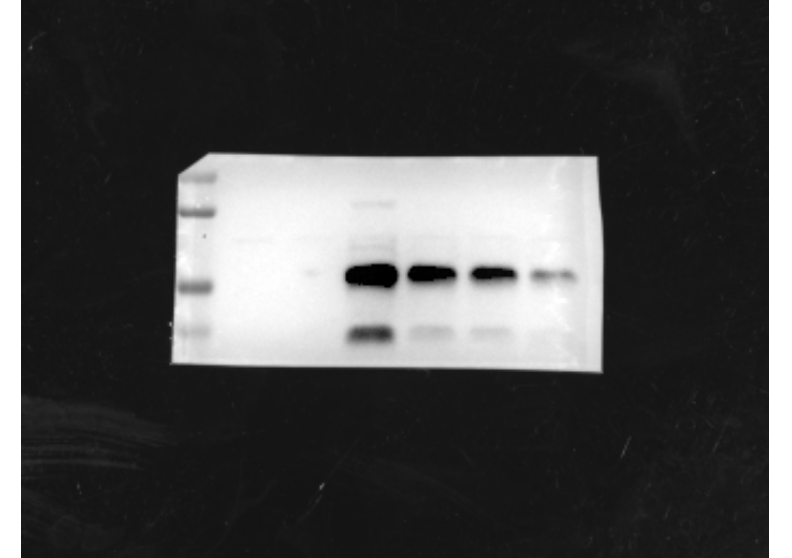
MCF7**

55 kDa

34 kDa

70 kDa

25 kDa

HA-HNRNPA1

C

d

c

b

a

EV


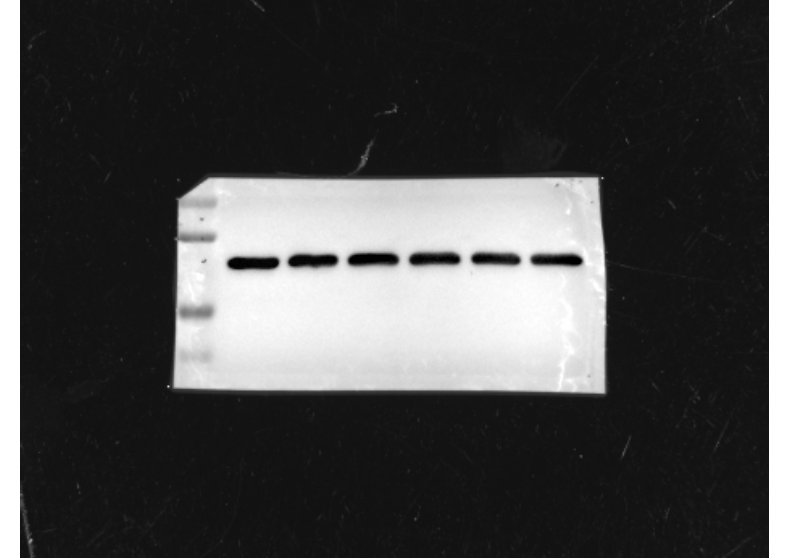


55 kDa

34 kDa

70 kDa

25 kDa

ACTB

**Replicate 1: MDA-MB-231** – HA-HNRNPA1 Western Blot image

EV

a

b

d

c

C


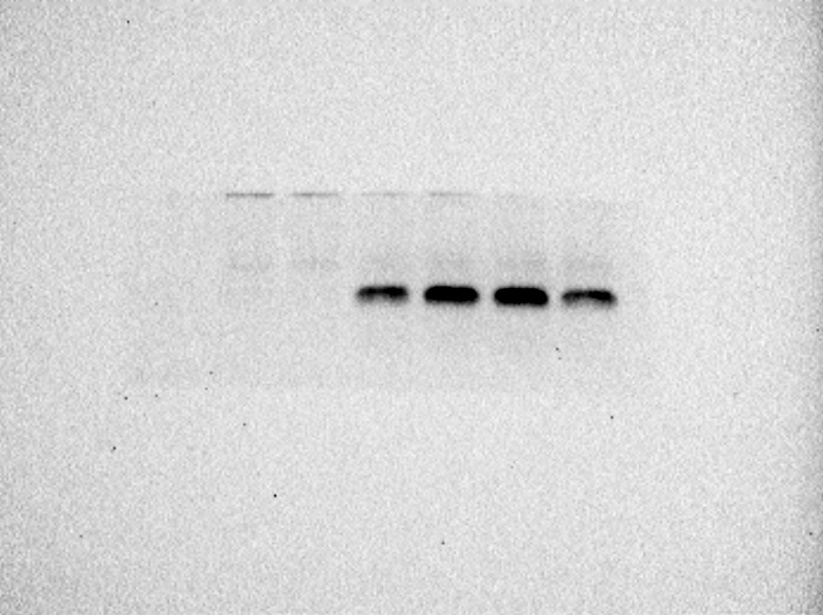


34 kDa

HA-HNRNPA1

MDA-MB-231 – ACTB Western Blot image (same blot)

EV

C

a

b

c

d


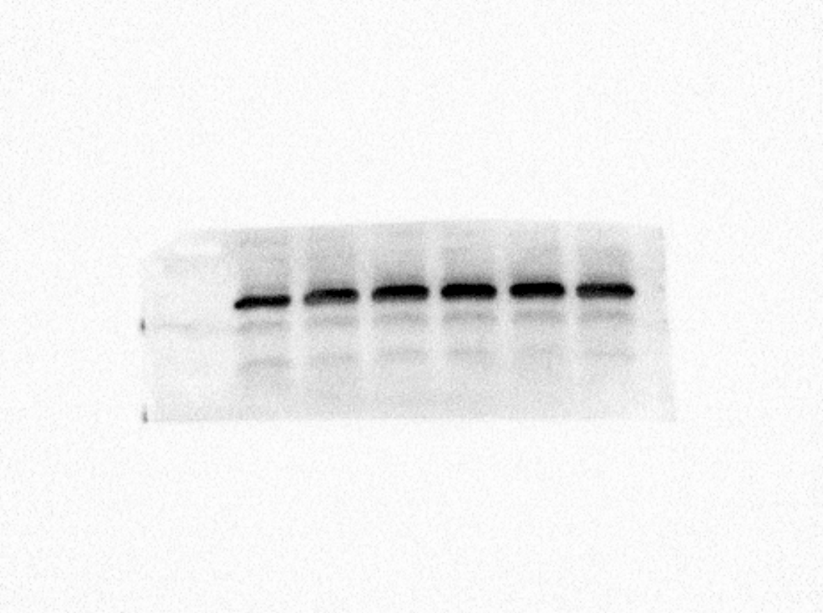


55 kDa

ACTB

**Replicate 2:**

**MDA-MB-231**

C

EV

c

b

a

d

**
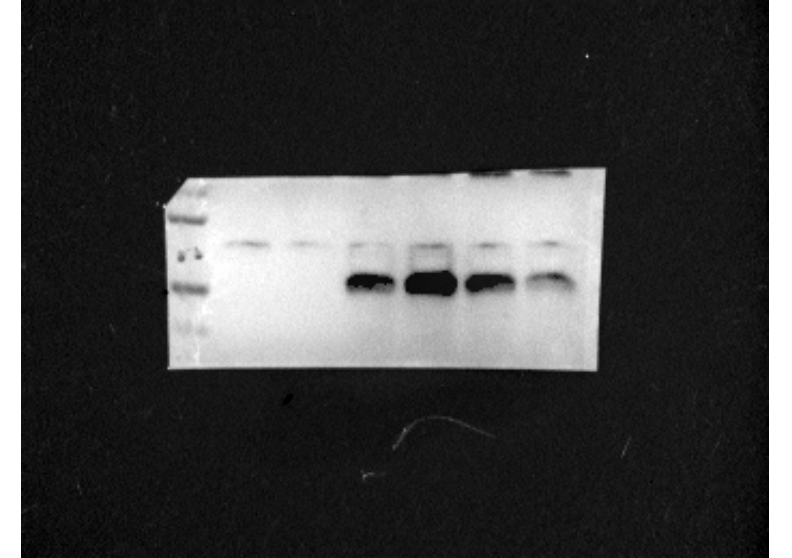
**

55 kDa

34 kDa

HNRNPA1

d

EV

a

b

c

C


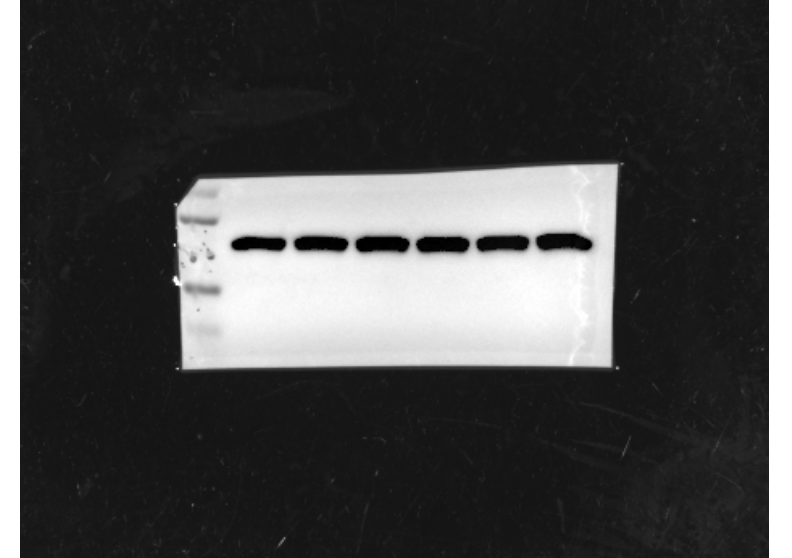


34 kDa

55 kDa

ACTB

**Replicate 3:**

**MDA-MB-231**

**
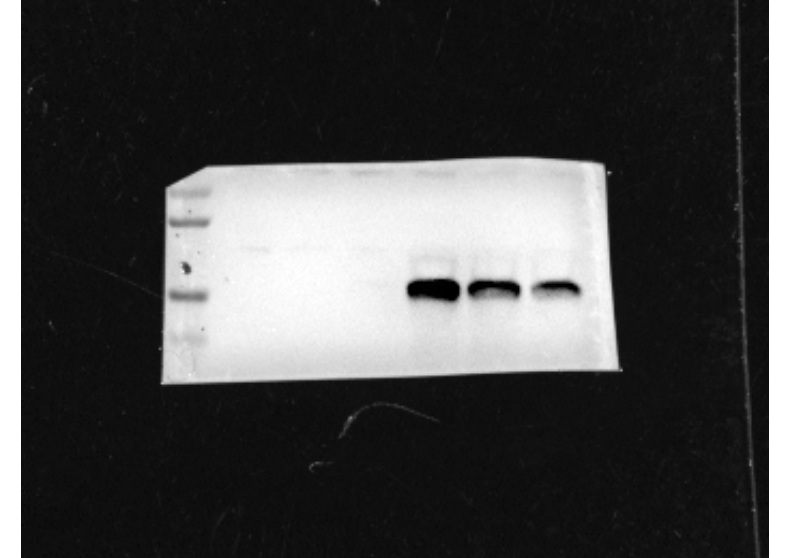
**

a*

C

EV

b

c

d

c

b

a

C

EV

55 kDa

HNRNPA1

34 kDa

**
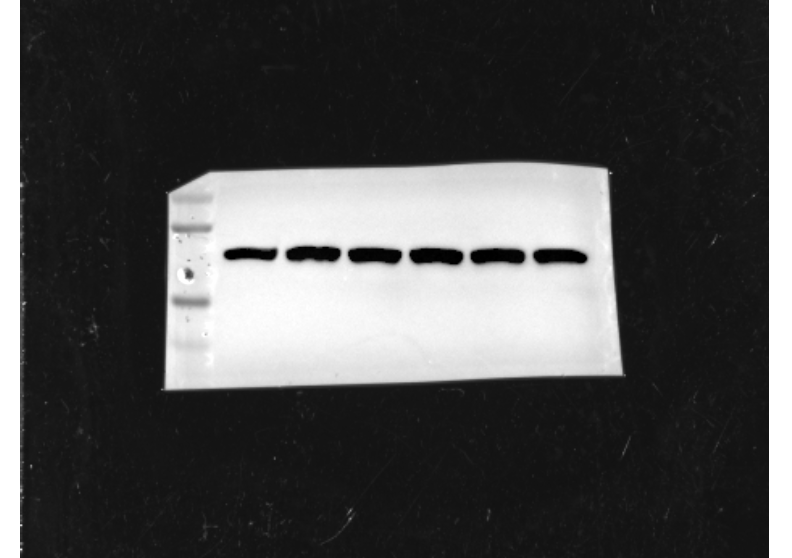
**

d

55 kDa

ACTB

34 kDa

*The transfection was not successful in this sample. Hence there are two values for the densitometric quantification of the HNRNPA1 band for the lane “a” in Fig.3C (for MDA-MB-231).

**Western Blot images for Fig. 5D**

MCF7 - HNRNPA1-sh Western Blot images. Blue dashed boxes show the presented data in the Fig. 5D. Independently isolated three sample sets were run side by side.

sh2

sh11

sh2

sh11

NT

NT

sh11

NT

sh2


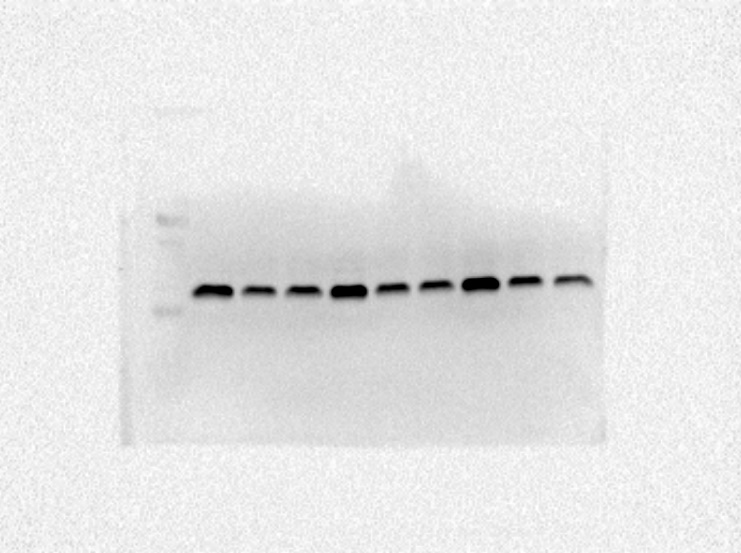


HNRNPA1

34 kDa

MCF7 - ACTB Western Blot image (same blot)

NT

sh2

sh2

sh11

sh2

sh11

NT

sh11

NT


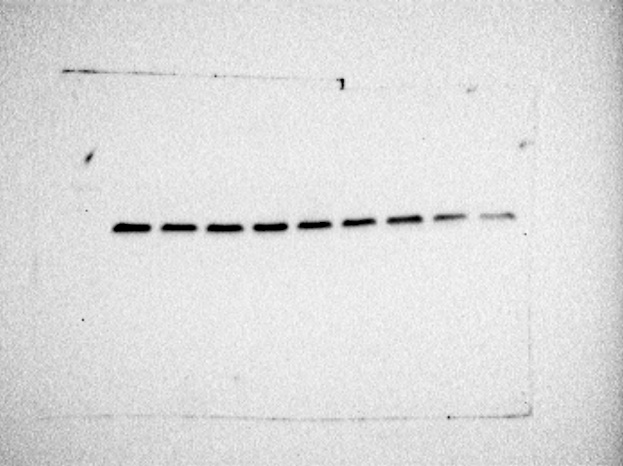


ACTB

42 kDa

MDA-MB-231 - HNRNPA1-sh Western Blot image

NT

sh11

sh2

NT

sh11

sh2

NT

sh11

sh2


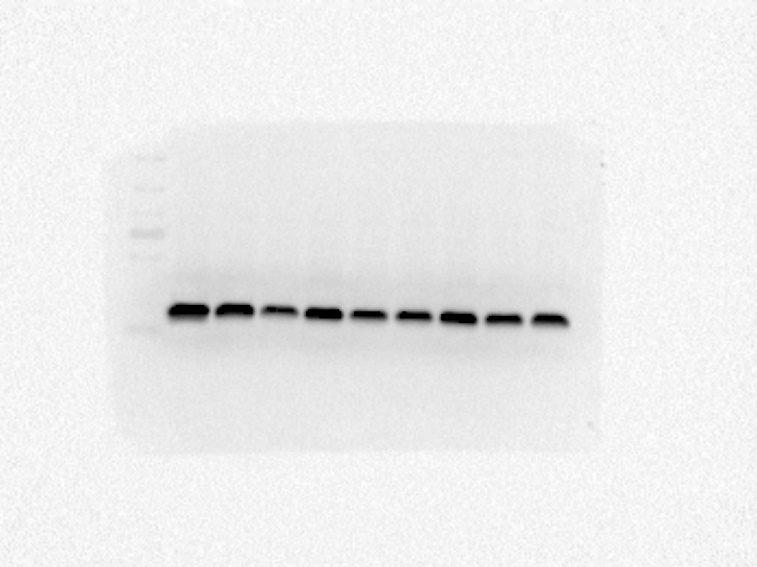


34 kDa

HNRNPA1

MDA-MB-231 - ACTB Western Blot image (same blot)

ACTB


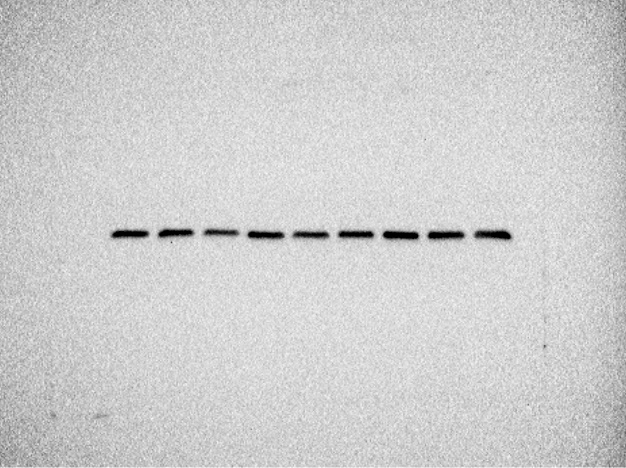


NT

sh2

sh11

NT

sh2

sh11

sh11

42 kDa

NT

sh2

**Wound Healing Images for Fig. 5G (MCF7)**

sh2

sh1

NT


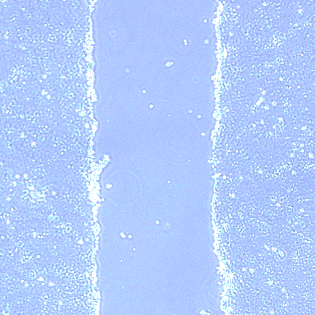

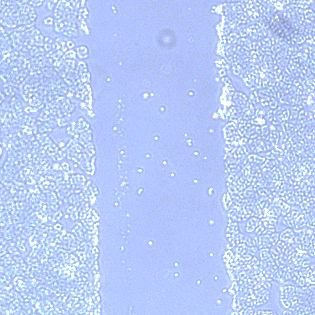

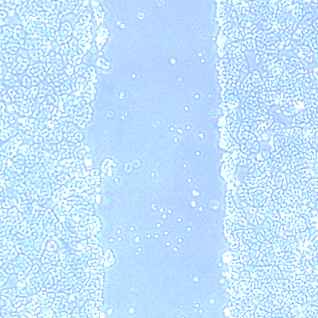


Day 0


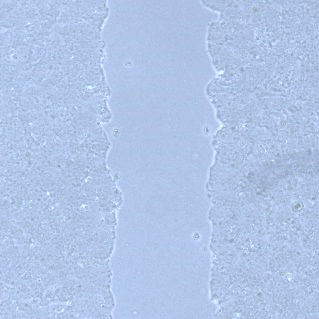

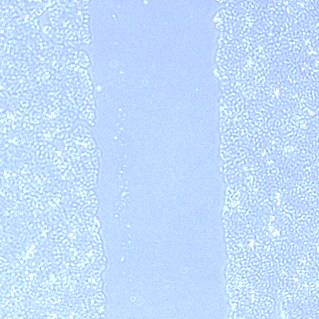

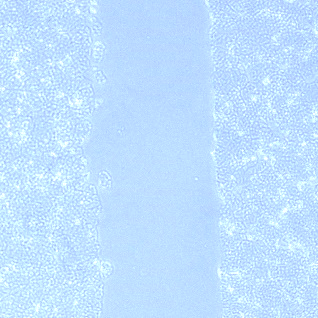


Day 1


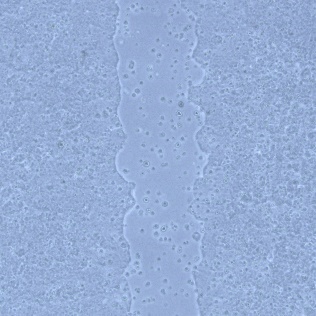

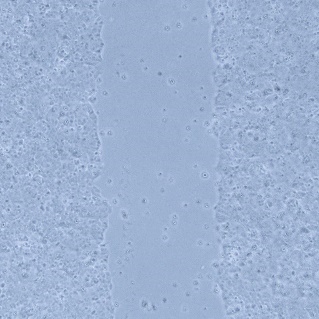

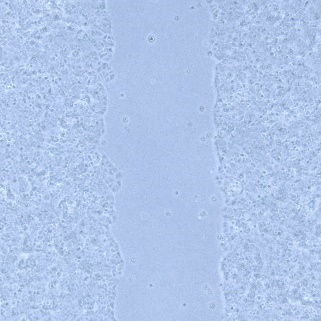


Day 2


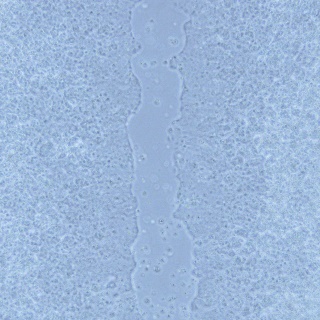

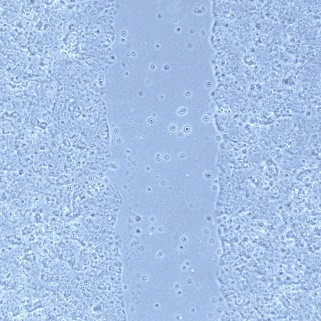

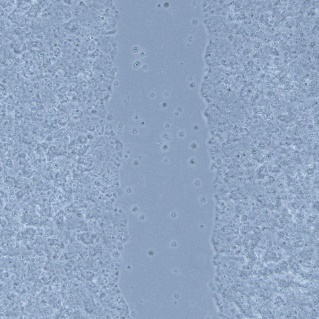


Day 3

**Wound Healing Images for Fig. 5G (MDA-MB-231)**

Day 0

Day 1

Day 2

NT

sh1

sh2

**
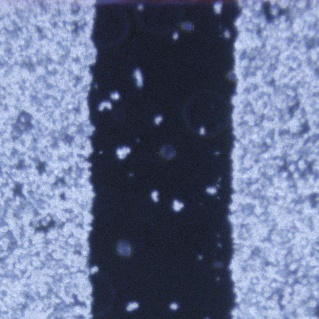

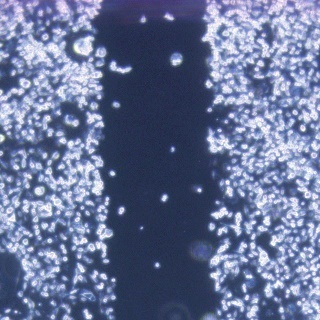

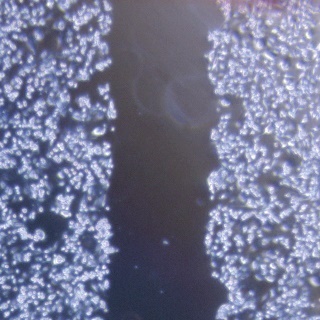
**

**
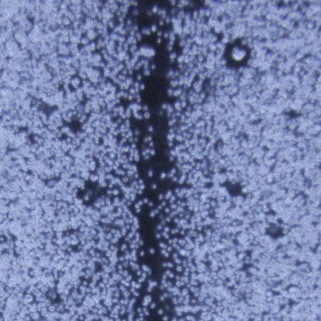

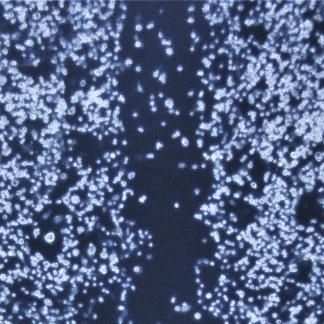

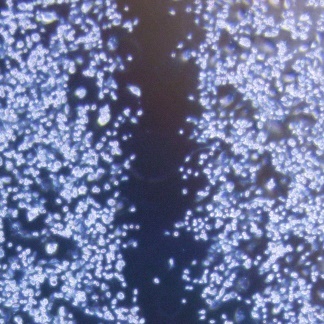
**

**
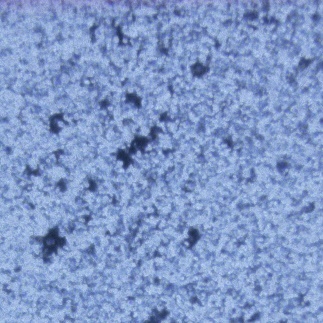

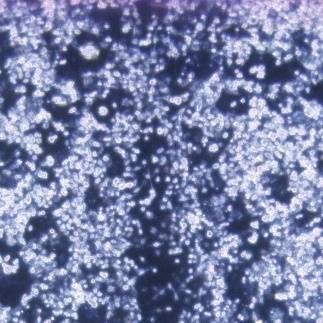

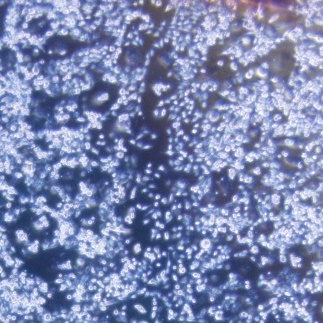
**

**Supplementary Tables**

**Supplementary Table S1.** Human gene-specific RT-PCR primer sequences.

| Name | Primer | Sequence (5’ to 3’) | Size | Temp. |
| --- | --- | --- | --- | --- |
| HNRNPA1 (NM_002136.4) | HNRNPA1 Isoform 2 F | AAGTGTAAAGCATTCCAACAAAGG | 98bp | 60^0^C  |
|  | HNRNPA1 Isoform 2 R | TCAGCGTCACGATCAGACTG |  |  |
|  | HNRNPA1  Isoform 3 F | CAACCTGCTTGGGTGGAGAA | 154bp | 60^0^C |
|  | HNRNPA1 Isoform 3 R | TTGCATAGGATGTGCCAACAA |  |  |
| HNRNPA1 (NR_104427.1) | HNRNPA1 Isoform 1 F | CAGAAGCTCTGGCCCCTATG | 203bp | 64^0^C |
|  | HNRNPA1 Isoform 1 R | CACAAGGACGTTCCTTTCTGC |  |  |
| RPLP0 (NM_001002.4 & NM_053275.3) | RPLP0 F | GGAGAAACTGCTGCCTCATA | 191bp | 59^0^C |
|  | RPLP0 R | GGAAAAAGGAGGTCTTCTCG |  |  |
| MYC  (NM_002467.6 & NM_001354870.1) | MYC F | CAGCTGCTTAGACGCTGGATT | 131bp | 61^0^C |
|  | MYC R | GTAGAAATACGGCTGCACCGA |  |  |
| VMP1 | Exon7 F | ATCGGTACAGCAATCGGAGA | 119bp | 59^0^C |
|  | Exon7 R | TCTGCATGTTCCAGCATCTCT |  |  |
| pri-miR-21 | F | TCGTGACATCTCCATGGCT | 199bp | 59^0^C |
|  | R | CCAGACAGAAGGACCAGAGT |  |  |
| C9ORF3(AOPEP) | Exon2-3 F | GAAGACAGGGGCTCAGACAG | 150bp | 60^0^C |
|  | Exon2-3 R | GCCCTGTTGTTTATGGGAGA |  |  |
| pri-miR-27b | F | ACCAGCTGAGGAAGATGCTC | 181bp | 62^0^C |
|  | R | CAGCGGCTCCAACTTAACTG |  |  |
| XIST | XIST F | TTACTCTCTCGGGGCTGGAA | 183bp | 560C |
|  | XIST R | GGAGGACGTGTCAAGAAGACA |  |  |

**Supplementary Table S2. Cloning primers**

| Name | Primer | Sequence (5’-3’) |
| --- | --- | --- |
| HNRNPA1 (NM_002136.4) | HNRNPA1 Isoform 2 F | CGCATTCTAGAAAAATGTACCCATACGATGTTCCAGATTACGCTTCTAAGTCAGAGTCTCCTAAAG |
|  | HNRNPA1 Isoform 2 R | CGCATGGATCCTTTACACAGCACATTAAAAAAAAGAC |
|  | HNRNPA1  Isoform 3 F | CGCATTCTAGAAAAATGTACCCATACGATGTTCCAGATTACGCTTCTAAGTCAGAGTCTCCTAAAG |
|  | HNRNPA1 Isoform 3 R | CGCATGGATCCTTCAAGAGAATTAAATCGTTTATTGATTAC |
| HNRNPA1 (NR_104427.1) | HNRNPA1 Isoform 1 F | CGCATTCTAGAAAAATGTACCCATACGATGTTCCAGATTACGCTTCTAAGTCAGAGTCTCCTAAAG |
|  | HNRNPA1 Isoform 1 R | CGCATGGATCCCTAAGTTAAATACAGTTTATTAAA |
| HNRNPA1 (CDS; NM_002136.4 & NR_104427.1) | HNRNPA1 CDS F | CGCATTCTAGAAAAATGTACCCATACGATGTTCCAGATTACGCTTCTAAGTCAGAGTCTCCTAAAG |
|  | HNRNPA1 CDS R | CGCATAAGCTTTTAAAATCTTCTGCCACTGCCATAG |
| HNRNPA1 shRNA sequences | Oligo seq1 | GATCCCCCGGAAACCTTGGTGTAGTTTTCAAGAGAAACTACACCAAGGTTTCCGTTTTTA |
|  | Oligo seq2 | AGCTTAAAAACGGAAACCTTGGTGTAGTTTCTCTTGAAAACTACACCAAGGTTTCCGGGG |

**Supplementary Table S3.** Primer sequences for the 3’UTRs of HNRNPA1 isoforms.

| Name | Primer | Sequence (5’-3’) |
| --- | --- | --- |
| HNRNPA1 (NM_002136.4) | HNRNPA1 S-3’UTR F | CGCATACTAGTTTAGGAAACAAAGCTTAGCAGG |
|  | HNRNPA1 S-3’UTR R | CGCATGAGCTCTTTACACAGCACATTAAAAAAAAGAC |
|  | HNRNPA1  L-3’UTR F | CGCATACTAGTTTAGGAAACAAAGCTTAGCAGG |
|  | HNRNPA1 L-3’UTR R | CGCATGAGCTCTTCAAGAGAATTAAATCGTTTATTGATTAC |
| HNRNPA1 (NR_104427.1) | HNRNPA1 Iso-1-3’UTR F | GAGCTCTTAGGGAGGAGTCTGCTACT |
|  | HNRNPA1 Iso-1-3’UTR R | AAGCTTCTAAGTTAAATACAGTTTATTAAAA |

**Supplementary Table S4**. Accession numbers for GEO datasets

| **GEO ID** | **Molecular Type** | **SRA Accession Number** |
| --- | --- | --- |
| GSE75688 (Expression profiling by high throughput single cell sequencing) | ER Positive | SRR2973272 |
|  | ER Positive | SRR2973280 |
|  | ER Positive | SRR2973282 |
|  | ER Positive | SRR2973279 |
|  | HER2 Positive | SRR2973274 |
|  | HER2 Positive | SRR2973301 |
|  | HER2 Positive | SRR2973302 |
|  | HER2 Positive | SRR2973303 |
|  | ER, HER2 Positive | SRR2973275 |
|  | ER, HER2 Positive | SRR2973276 |
|  | Triple Negative | SRR2973277 |
|  | Triple Negative | SRR2973278 |
| GSE113197 (Expression profiling by high throughput single cell sequencing) | Breast Epithelium-Basal | SRR7008472 |
|  | Breast Epithelium-Basal | SRR7008502 |
|  | Breast Epithelium-Basal | SRR7008484 |
|  | Breast Epithelium-Luminal | SRR7008518 |
|  | Breast Epithelium-Luminal | SRR7008527 |
| GSE10797, GSE20437, GSE9574, GSE6883, GSE26910, GSE21422, GSE3744 and GSE2361 (Expression profiling by array) | Normal Breast Samples |  |
| GSE31519, GSE2034, GSE7390 (Expression profiling by array) | Breast Cancer Patient Samples |  |

Reference:

Nathan W Wong, Yuhao Chen, Shuai Chen, Xiaowei Wang,  *OncomiR*: an online resource for exploring pan-cancer microRNA dysregulation, *Bioinformatics*, Volume 34, Issue 4, 15 February 2018, 713–715.
